# Boosting supercapacitor performance through innovative transition metal-based electrode materials

**DOI:** 10.1039/d5ra02905h

**Published:** 2025-09-22

**Authors:** Ahmed Alharbi

**Affiliations:** a Department of Chemistry, Faculty of Science, Umm Al-Qura University Makkah Saudi Arabia amaharbi@uqu.edu.sa

## Abstract

Transition metal-based electrode materials—particularly oxides (TMOs) and sulfides (TMSs)—have emerged as pivotal candidates for enhancing supercapacitor (SC) performance, addressing critical limitations in energy density, power density, and cycle stability. This comprehensive review systematically explores recent advancements in innovative TMO electrodes, including MnO_2_, NiO, ZnO, Co_3_O_4_, VO_*x*_, and RuO_2_, and TMS electrodes including binary/ternary sulfides (*e.g.*, NiCo_2_S_4_ and CoMoS_4_). Key fabrication techniques, such as sol–gel processing, electrodeposition, hydrothermal synthesis, and chemical vapor deposition, are evaluated for their role in tailoring the material morphologies (*e.g.*, nanosheets and core–shell heterostructures) and optimizing the electrochemical properties. The synergistic effects in hybrid composites (*e.g.*, rGO/NiO-Mn_2_O_3_ and CNT@MnO_2_) significantly enhance the conductivity, ion diffusion, and faradaic redox activity, achieving remarkable specific capacitance (up to 1529 F g^−1^ for ZnO@Ni_3_S_2_) and retention rates (*e.g.*, 91% over 500 cycles for NiO-Mn_2_O_3_@rGO). This review further contrasts TMOs and TMSs, highlighting the latter's superior electrical conductivity and reversible kinetics while noting the challenges in synthesis scalability and stability. Critical challenges, including low energy density, manufacturing costs, and industrial standardization, are discussed alongside future directions, such as flexible/wearable SCs, intelligent devices, and sustainable material design. This work underscores the transformative potential of transition metal-based electrodes in bridging the performance gap between capacitors and batteries, paving the way for next-generation energy storage systems.

## Abstract 2

Transition metal oxides (TMOs) and sulfides (TMSs) are key electrode materials. These materials are promising for supercapacitors (SCs). They can improve SCs' performance.

## Graphical abstract

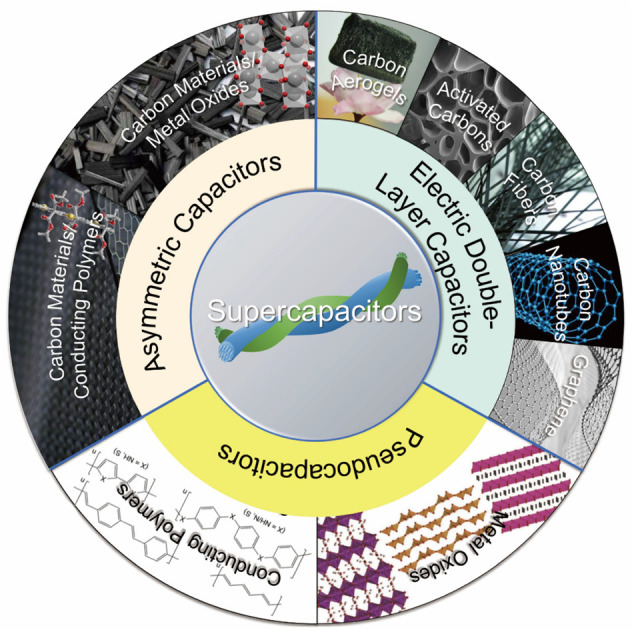

## 1. Introduction

The continued development and use of natural resources, such as oil, natural gas, and coal, are essential for the growth of global economy. There are concerns about resource sustainability and exhaustion due to the increasing use of these assets.^1–6^ Accessible and ecologically friendly energy is one of the Sustainable Development Goals (SDGs) set out by the United Nations Environment Programme (UNEP). By 2020, fossil fuels accounted for 84.3% of the global power use, which increased greenhouse gas emissions and exacerbated global warming.^7^ Efforts have been made to accelerate the research and development of other sources of energy, such as biomass, tidal energy, and wind, in order to solve these issues.^8^ Consequently, energy storage systems that are secure, safe, and efficient are essential to easily store the energy generated by these new resources.^9^ Energy storage devices are categorized into several types, with SCs and batteries being two examples.^10^

**Fig. 1:**
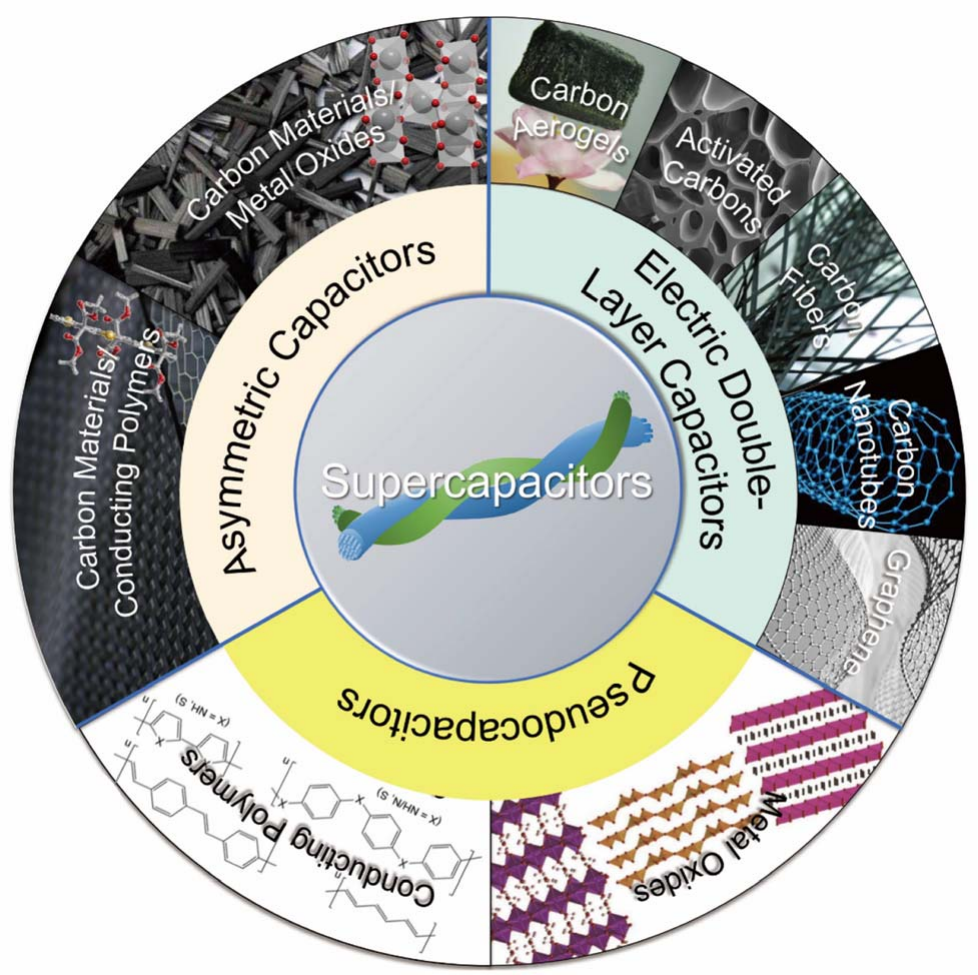
Schematic of the commonly used electrode materials for various supercapacitor types. Asymmetric capacitors combine carbon materials with conducting polymers or metal oxides, while pseudocapacitors utilize metal oxides and conducting polymers. Graphene, carbon aerogels, activated carbons, carbon fibers, and carbon nanotubes are predominantly employed in EDLCs.^31^

Energy storage systems are categorized in two ways. These categories are based on how they store energy: batteries and SCs. Compared to batteries, SCs provide significant benefits in terms of cycle stability and power density. The energy and power densities of SCs may vary significantly over orders of magnitude with careful design, making them a flexible option for energy storage.^10–13^ Supercapacitors, also known as electrochemical capacitors, ultracapacitors, or supercap batteries, are a class of energy storage devices delivering outstanding performances. By utilizing several energy storage methods, including redox reactions, physical adsorption, electrochemical double layer capacitance, and pseudocapacitance, they enable efficient energy storage and release.^14^Table 1 presents a comparison among the different types of storage mechanisms of supercapacitors. The current drawbacks of lithium-ion batteries, which include problems with electron and lithium-ion transport in the electrodes, polarization effects, charge transport across phase boundaries, and slow redox reactions, make it difficult for them to store energy effectively at rapid rates. Furthermore, these difficulties cause a large amount of heat to be produced throughout the charging process.^15^ A detailed comparison between SCs, regular capacitors and batteries is presented in Table 2. Moreover, the low cycle life of lithium-ion batteries indicates a shorter lifespan, requiring replacements every three to five years and resulting in increased maintenance costs. SCs, however, have a higher power density (103–105 W kg^−1^), longer cyclic life (1000–100 000 cycles), safeguarding, and incredibly quick charging and discharging periods (a few seconds). This is explained by the notion that physical processes constitute the majority of energy storage methods.^9,16–18^ Additionally, because of their long life cycle, high power density (*P*_d_), high specific capacitance (*C*_sp_), minimal upkeep needs, lack of memory effect, security characteristics, and ability to act as a bridge to bridge the power-energy gap between fuel cells/batteries (large energy storage) and capacitors (high *P*_d_), SCs have attracted much attention.^19–22^ However, the reason for the restricted use of SCs in appliances and gadgets is their low energy density (*E*_d_) (5–10 W h kg^−1^). Scientists have proposed hybrid SCs as a solution to this discrepancy between batteries and supercapacitors. The goal of these hybrids is to combine the greater power density and longer cycle life of SCs with the high *E*_d_ of the batteries. This is accomplished by combining a Faradaic electrode and a capacitive electrode (Fig. 1).^23^

**Table 1:** Comparison among hybrid supercapacitors, pseudocapacitors, and EDLCs.^24^

EDLC	Pseudocapacitor	Hybrid capacitor
Carbon is used to make the electrodes	Conductive polymers and metal oxides are used to make the electrodes	A mix of carbon, conductive polymers and metal oxides is used
Charge storage proceeds through the non-faradaic mechanism of electrochemical double layer production	Charge storage is a faradaic process since it occurs *via* redox processes	A mixture of faradaic and non-faradaic processes is involved in charge accumulation
Low *C*_sp_, dependable cycle stability, moderate *E*_d_, and excellent rate capability	Significant *E*_d_, high *C*_sp_, noteworthy *P*_d_, and restricted rate capabilities	Li/C capacitors are expensive, but polymer/carbon composites are moderately priced and stable. They have a high *E*_d_, significant *P*_d_, and dependable cyclability

**Table 2:** Comparison among SCs, regular capacitors and batteries according to ref. 47

Property	SCs	Capacitors	Batteries
*P*_d_ (W h kg^−1^)	1000–2000	>10 000	<1000
*E*_d_ (W h kg^−1^)	1–10	<0.1	10–100
Recharge time (s)	1–30	10^−6^ to 10^−3^	1080–10 800
Discharge time (s)	1–30	10^−6^ to 10^−3^	3600–18 000
Recharge/discharge efficiency (%)	90–95%	100%	—
Cycle life (cycles)	>100 000	>500 000	<1000
Factors influencing stored charge	Microstructure of electrodes and the electrolyte	Area of electrode and dielectric	Mass of the active material and thermodynamic considerations
Factors influencing the maximum voltage	Stability range of the electrode and electrolyte	Thickness and strength of the dielectric	Thermodynamic aspects of phase reactions

Because of their large and rigid constructions, conventional capacitors are believed to be unsuited for use in future applications. Transparent, flexible, lighter, and thinner supercapacitors with a range of cutting-edge properties are in high demand. These are necessary for creating consumer devices with many uses. Carbon nanotubes (CNTs), transition metal oxides (TMOs) and activated carbons (ACs) are the most common materials used in electrodes nowadays. Wide holes, poorly graphitized frameworks, and uneven morphologies are characteristics of AC. TMOs, however, have limited electronic conductivity, making them less appropriate for high-rate energy storage situations.^25^ As a result, scientists have been investigating new materials for SC electrodes, such as mixed conductors, metal sulfides, 2D materials, covalent organic frameworks (COFs), metal–organic frameworks (MOFs), metal nitrides, and MXenes.^26–28^ SCs can be divided into three configuration types: asymmetric supercapacitor (ASC) (using two different types of electrode materials with the same energy storage technique), symmetric supercapacitor (SSC) (using two types of electrode materials), and asymmetric hybrid supercapacitor (AHSC) (using two types of electrode materials with different energy storage techniques). Currently, AHSCs are widely used for increased energy storage efficiency.^29^

SCs have enormous promise for many commonplace uses, such as power supply, cameras, digital communication equipment, electric hybrid cars, and cell phones. Combined with batteries, SCs may greatly increase the performance of hybrid electric vehicles (HEVs) by assisting with recovering energy, allowing for effective charging under cold conditions, enabling strong acceleration, and prolonging the life of batteries. Furthermore, SCs provide excellent integration and interoperability with HEVs and metro rail systems.^30–36^ For example, several research groups have recently published data on various types of multifunctional SCs. SCs that are flexible, self-healing, electrochromic, and self-charging are all included in these studies.^37^ These sophisticated multipurpose SCs can refuel on their own, human motion, employing heat, solar energy and several types of mechanically deformed energy. Their potential for incorporation into wearable and mobile electronic goods has been improved through this characteristic.^38,39^ These provide a workable option for energy delivery in remote areas without public grids or in circumstances in which installing electrical infrastructure would be expensive. Because SC is small, light, and flexible, it may also be used as an energy source for handheld devices, such as cameras, notebooks, and cell phones. SCs may supply the high *P*_d_ required for quick acceleration in electric and hybrid cars. Energy savings and battery protection from frequent interruptions are achieved through energy recovery during the charge and discharge cycles inherent in dynamic operations.^40,42^

Although lithium-ion batteries (LIBs) can achieve remarkable densities of energy around 180 W h kg^−1^, their power supply and absorption are usually somewhat slow.^41^ As a result, the extensive use of LIBs faces significant limitations, particularly in energy storage systems that demand rapid and high-power storage capabilities.^41–44^ Consequently, SCs have taken on the duty of tackling this significant obstacle.^45^Table 3 illustrates a comparison between different types of supercapacitors and batteries. Currently, bridging the power gap between batteries and traditional electrolytic and solid-state capacitors critically depends on SCs. Compared to batteries, they offer greater power bursts; compared to ordinary capacitors, they store more energy. Although the *E*_d_ of the majority of readily accessible SCs has a lower value than that of batteries and fuel cells (less than 10 W h kg^−1^), it is still higher than that of ordinary dielectric capacitors.^46^ With the world economy expanding so quickly, there is an increasing need for SCs with high energy storage. Significant research efforts have been made to improve the *E*_d_ of SCs despite preserving their high-power capacity to equal or surpass that of batteries. Recent years have witnessed a concerted global push to decrease manufacturing expenses.^47–50^

**Table 3:** Advantages and disadvantages of sodium-ion batteries and supercapacitors.^48–52^

Device	Advantage	Disadvantage
SC	Elevated power density	Low *E*_d_
	Extended cycle life	
	Rapid rate of charging	
	Excellent performance at low temperatures	
	High capacity to discharge current	
	Features of extremely low temperatures	
	Basic circuit for charging and draining	
	Easy detection	
Sodium-ion battery	Equivalent *E*_d_ to that of a lithium-ion battery	Compared to lithium-ion batteries, sodium-ion batteries have a greater technical threshold
	High in sodium	Comparing the overall electrochemical performance to lithium-ion batteries, it is inferior
	Robust stability	It is unclear how hard carbon is stored
	Elevated safety	
	Extended lifespan	
	Numerous uses	
	Simple availability of raw ingredients	
	Waste recycling is an easy, non-polluting procedure with quick charge and discharge. Because sodium ion batteries do not have over discharge properties, they can discharge to 0 V	

## 2. Tools and assessment of vital parameters

A range of techniques and instruments have assessed the electrochemical efficacy of SCs. These include constant current charge/discharge (CCCD), cyclic voltammetry, and electrochemical impedance spectroscopy (EIS). All of them measure voltage, current, and time, but they can also be used to determine other characteristics, including power performance, energy, time constant, operating voltage, capacitance, and equivalent series resistance. We further investigate the unique priorities and limits of each instrument.

### 2.1. EIS

The electrochemical system comprises a working electrode (WE), a counter electrode (CE), and a reference electrode (RE). At point (a), the current generated by applying the potential, *E*(*t*), between the WE and RE is measured. (b) A periodic perturbation signal with an amplitude (Δ*E*) is applied between the WE and RE, transitioning from high to low frequencies. (c) The electrochemical response to this perturbation is quantified within the linear domain. (d and e) Impedance data are typically presented using Nyquist or Bode plots (part d) and can be modeled by an equivalent circuit. This circuit allows for a mechanistic interpretation of the system, which helps in extracting the meaning of the faradaic impedance, *Z*_F_ (part e). Key terms include *α* (parameter associated with the CPE), *ω* (angular frequency), CPEdl (double-layer constant-phase element), EIS (electrochemical impedance spectroscopy), *f* (frequency), *ϕ* (phase angle), *Q* (parameter associated with the CPE), Re (ohmic resistance), *Z*_j_ (imaginary part of the impedance), and *Z*_r_ (real part of the impedance).^53^

**Fig. 2:**
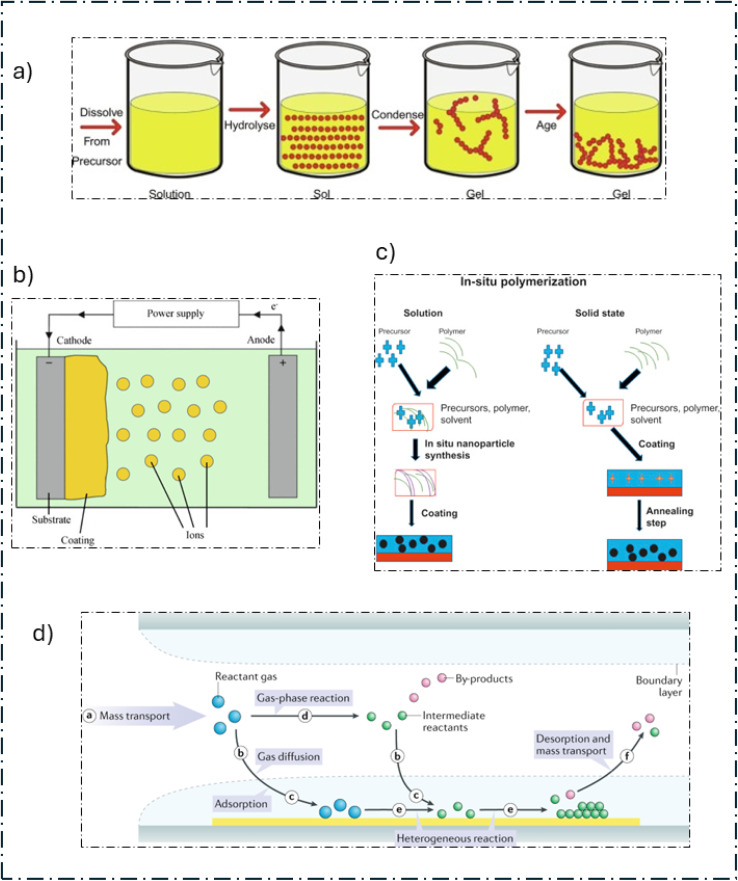
(a) Sol–gel technique,^65^ (b) electrodeposition technique,^66^ (c) *in situ* polymerization,^67^ and (d) chemical vapor deposition (CVD).^68^

EIS, or dielectric spectroscopy, uses a small AC voltage (around 5 mV) added to a DC potential to measure a battery's impedance at various frequencies (Fig. 2). Visual representations of the resulting data frequently include Nyquist plots (imaginary and real cell impedance components on a complex plane) and Bode plots (cell response across phase angle and frequency).^58,59^

EIS has been utilized to determine the characteristics of capacitance, energy, and power, in addition to the impedance and frequency response. It was additionally employed to evaluate the processes of mass transport, charge storage and charge transfer.^54,55^ To discern how each structural element in a cell system contributes to the overall impedance, many analogous circuitries and concepts have been created.^56–58^ Equivalent series resistance (ESR) in superconductor device assessments is often represented in the literature by impedance components at particular frequencies. However, it is important to remember that the *R*_ES_ obtained from an EIS test is frequently far less than the *R*_ES_ obtained from a CCCD analysis, and as a result, it cannot fully capture the power efficiency of SC devices.^59^ By performing a comparable analysis in a three-electrode setup, EIS testing may be utilized to investigate the charge transfer, impedance, *C*_sp_, charge storage and mass transport techniques associated with SC materials.

### 2.2. CV

Applying a progressively varying electric voltage over two or three electrode configurations is the process of CV testing. Controllable parameters include the potential window and the rate of potential change (sweep rate, also known as scan rate, *v*). Electrochemical reactions may be observed by the current that results from cathodic and anodic sweeps. Plotting data usually show current against potential, but it may also show current or potential against time.^60,61^

To differentiate between the pseudocapacitor and EDLC behavior in SC materials, three-electrode CV testing offers the most suitable charge storage investigation method.^68,69^ An initial interpretation of the test results can be obtained from the shapes of the CV curves. EDLC and most pseudocapacitors produce rectangular CV curves; however, some materials show highly reversible redox peaks in their CV curves.^70^ Thus, based only on the CV diagram form, it is insufficient to distinguish between EDLC and pseudocapacitor materials.

A more reliable quantitative method for distinguishing EDL and pseudocapacitor contributions in CV data is to assume that the EDL current is directly proportional to the scan rate. Conversely, the rate of diffusion-limited cation adsorption/insertion at the electrode surface for the pseudocapacitor mechanism is proportional to the square root of the scan rate.^71–77^ Meanwhile, surface-redox actions and EDL processes occur essentially on the same time scale; this technique is restricted in its capacity to distinguish between their contributions.^61^ To solve this issue, additional research, both theoretical and experimental, is needed.

In practice, the potential window and operating voltage for SC materials are determined *via* CV testing with a three-electrode setup, where the reversal potential is gradually adjusted. Continuous study of the reversibility of both charging and discharging operations is also possible.^62,63^ Furthermore, the integration of the CV diagrams can provide the *C*_sp_ and energy efficiency of the SC materials. For SC devices, a similar procedure may be used to determine the total cell capacitance and, consequently, the quantity of stored electricity.

### 2.3. CCCD

CCCD analysis is a common technique for characterizing SCs with a direct current. Typically, the experiment yields a graph of potential (*E*) *versus* time (s) from repeatedly charging/discharging the SC/electrode at a constant current; the interval between charge and discharge does not affect the constant peak voltage (*V*_o_). For a CCCD test to yield comparable and uniform results, the steady current level must be set correctly.^64^

The CCCD experiment is considered a reliable and adaptable method for assessing SC. It is possible to examine the three fundamental variables in SC devices—total cell capacitance (*C*_T_), *R*_ES_, and *V*_o_—from which the most additional characteristics, comprising the time constant, energy and power densities, leakage, and peak current, may be obtained. It is also applied to investigate the cycle stability of SC devices. Additionally, the CCCD test may be used to determine the particular reversibility, potential, and capacitance window for SC employing a three-electrode configuration.

## 3. Electrode material fabrication techniques

The architecture and characteristics of electrode materials are greatly influenced by the method of creation. A summary of many synthesis techniques is provided below.

### 3.1. Sol–gel technique

Sol–gel processing (Fig. 2a) offers a straightforward approach to creating materials with enhanced homogeneity and purity. This process entails the controlled aggregation and joining of microparticles within a solution (sol) to create a cohesive network (gel). There are two primary methods—colloidal and polymeric/alkoxide—differentiated by their precursor materials. In both methods, the precursor is combined with a liquid (alcohol for polymeric materials and water for colloidal materials), and an acid or base is used to activate the mixture. After going through a reaction to create a network, the activated precursor gradually fills the container as it grows older and more stable with temperature and time.^69^ Many TMOs have been prepared using this technique. The ability to produce materials with different morphologies is one of its advantages. With surfactant, solvent, reaction time, temperature modification, and enhanced electrochemical efficacy, the resultant electrode material has a high specific surface area.^70^

By employing this technique, Yusin and collaborators^71^ produced a composite of activated carbon fiber material (ACFM) and Ni(OH)_2_ with a *C*_sp_ of around 370–380 F g^−1^. Additionally, they determined how the volume, form, and structure of the material were affected by the concentration and composition of the solution. Furthermore, Liu and colleagues^72^ used a sol–gel method to create NiCo_2_O_4_ films, which produced high cycling stability.

### 3.2. Electrodeposition technique

Due to its capacity to accurately regulate both film thickness and polymerization rate, this synthetic process (Fig. 2b) is frequently employed. It is possible to create nanostructured films with different morphologies and mass loadings by choosing the appropriate deposition solution. This is noteworthy because it uses few harmful chemicals under simple production conditions. This process is usually used to prepare CPs such as polypyrrole (PPy), PEDOT, and polyaniline (PANI).

Electrochemical deposition was the method used by Poonam *et al.*^89^ to deposit stretchable CNT-PPy films. Electrodeposition by Ge *et al.*^90^ yielded ZnO@Ni_3_S_2_ core–shell nanorods; these nanorods displayed a *C*_sp_ of 1529 F g^−1^ at 2 A g^−1^, maintaining 42% of their original *C*_sp_ over 2000 cycles. By electrochemically growing nanosized MnO_2_ electrodes on Au nanowire stems, Chen and collaborators^73^ showed remarkable properties, such as durability over time (90% *C*_sp_ preservation after 5000 cycles) and a high *C*_sp_ of 1130 F g^−1^ at 2 mV s^−1^.

### 3.3. *In situ* polymerization

The sonication of monomers in an aqueous solution, followed by an oxidizing agent to initiate polymerization, is shown in Fig. 2c. Filtration of the solution yielded the final sample. At first, this procedure mostly created irregular aggregates that contained a trace amount of nanofibers. However, minor adjustments produced NPS, NRs, and nanofibers, which improved the physical and chemical characteristics of the solution and made it easier to treat.

*In situ* electro-polymerization was employed by Wang and colleagues^74^ to deposit PANI nanowires into multi-walled carbon nanotubes (MWCNTs). In addition to providing structural support for the organic polymers, the aligned MWCNTs act as channels for charge transfer. Furthermore, the structure's lifetime is increased by the constrained MWCNT channels, which prevent structural alterations in PANI chains throughout charge–discharge cycles. At 1.6 A g^−1^, films containing CPs enclosed in MWCNTs showed a *C*_sp_ of 296 F g^−1^. In another study, Zhou *et al.*^75^ used graphene and MWCNTs coupled with different π-conjugated sulfonate templates to examine the polymerization properties of PEDOT. The resultant PEDOT:MWCNT composite demonstrated a *C*_sp_ of 199 F g^−1^ at 0.5 A g^−1^, enabled by π–π interactions between PEDOT and non-covalently functionalized MWCNTs.

### 3.4. Direct coating

Using this technique, an active material liquid is immediately applied to a substrate to form SC electrodes. To guarantee good adhesion and preserve electrical conductivity, binders such as carbon black, polyvinylidene fluoride (PVDF), polytetrafluoroethylene (PTFE), and acetylene black are usually used.

Jana and collaborators^76^ mixed 10% PVDF and (*N*,*N*-dimethyl formamide) DMF with carbon fabric that had been processed with nitric acid to make an SC electrode slurry, which was subsequently applied to a stainless-steel platform. A SC electrode was created by Du and colleagues^77^ by smearing nickel foam (NF) with a slurry consisting of active material, PTFE and acetylene black.

### 3.5. CVD

The CVD process is usually used whenever porosity is important. As depicted in (Fig. 2d), the reactant gases (blue circles) enter the reactor (step a) in chemical vapor deposition (CVD). They either adsorb (step c) or diffuse directly to the substrate (step b) or react in the gas phase to create intermediates (green) and by-products (red) (step d). On the substrate, these intermediates are subsequently adsorbed (step c) and dispersed (step b). The formation of thin films or coatings is preceded by surface diffusion and reactions (step e). Finally, unreacted gases and byproducts are desorbed and released as exhaust (step f). This works in the vapor phase, where the substance is first vaporized and then heated to temperatures between 800 and 1000 °C. The structures that are produced have a homogeneous morphology.^78^ With its extensive crystal structures, single-layered structure, and reduced sheet defects, graphene produced *via* CVD offers superior results to graphene synthesized by applying other methods, such as chemical exfoliation, organic solvents, physical separation of graphite, reduction of graphene oxide (GO) from graphite oxidation, and arc discharge for multi-layered graphene. Having these qualities helps to increase carrier mobility.^79^

Utilizing graphene generated by CVD and hybridized with MWCNTs, Kalam and collaborators^80^ demonstrated the manufacture of outperforming SCs with increased electrochemical characteristics. In the meantime, Lobiak and colleagues^81^ created hybrid carbon materials using CVD on a metal catalyst supported by MgO, which included MWCNTs and graphitic layers. These substances enable quick charge transfer inside the cell.

The discussed fabrication techniques, sol–gel processing, electrodeposition, *in situ* polymerization, direct coating, and CVD, offer a versatile toolkit for engineering high-performance electrode materials tailored to specific electrochemical applications. The sol–gel technique stands out owing to its simplicity, excellent control over purity, and ability to tailor morphology through parameter tuning, yielding materials with high surface areas and promising specific capacitances. Electrodeposition, on the other hand, provides precise control over film thickness and morphology with environmentally benign processing, making it ideal for creating nanostructured films and composite electrodes with enhanced durability and performance. *In situ* polymerization facilitates the integration of conductive polymers into carbon-based scaffolds, improving mechanical stability and charge transport, particularly through π–π interactions and nanostructuring. The direct coating method offers a straightforward, scalable route for slurry-based electrode fabrication, relying on binders to ensure adhesion and conductivity, while CVD remains the method of choice for synthesizing uniform, defect-minimized carbon nanostructures, like graphene and MWCNTs, delivering exceptional electrochemical and structural properties. Collectively, these techniques demonstrate how tailoring material morphology, porosity, and interface chemistry can significantly enhance the performance and stability of electrode materials in supercapacitor applications.

Electrodeposition, though eco-friendly and inexpensive, is generally constrained by the need for conductive substrates and may result in uneven film growth if parameters such as current density or deposition time are not precisely controlled. Furthermore, for multi-component or layered materials, achieving uniform co-deposition can be challenging. The *in situ* polymerization technique shows significant promise in forming well-integrated composite materials, particularly conductive polymer-carbon hybrids. However, the morphology of the resulting structures is highly sensitive to synthesis conditions, such as monomer concentration, oxidant ratio, and sonication duration. Uncontrolled polymer growth may lead to agglomeration or poor dispersion, reducing electrochemical accessibility and mechanical stability over repeated cycles.

Direct coating remains appealing because of its simplicity and low-cost processing, especially in commercial supercapacitor fabrication. However, this method relies heavily on the use of binders (*e.g.*, PVDF and PTFE) and solvents (*e.g.*, DMF), which may introduce resistive interfaces and limit ion transport if not carefully optimized. Moreover, achieving uniform slurry distribution and strong adhesion on metallic or porous substrates remains a practical challenge. CVD, though highly effective for producing defect-free and crystalline carbon nanostructures, is one of the most energy- and cost-intensive techniques. Operating temperatures above 800 °C require advanced thermal management and reactor control, making the technique less viable for large-scale manufacturing. Additionally, CVD often demands metal catalysts and inert atmospheres, adding complexity and increasing production costs. Although the resulting materials (*e.g.*, graphene and MWCNTs) offer outstanding conductivity and structural integrity, the scalability and environmental footprint of CVD remain areas of concern.

The selection of a fabrication technique must strike a balance between material performance, process scalability, and environmental impact. Hybrid approaches, such as combining sol–gel or electrodeposition with CVD-grown nanostructures, may offer pathways to leverage the advantages of each method while mitigating individual drawbacks.

### 3.6. Vacuum filtration method

**Fig. 3:**
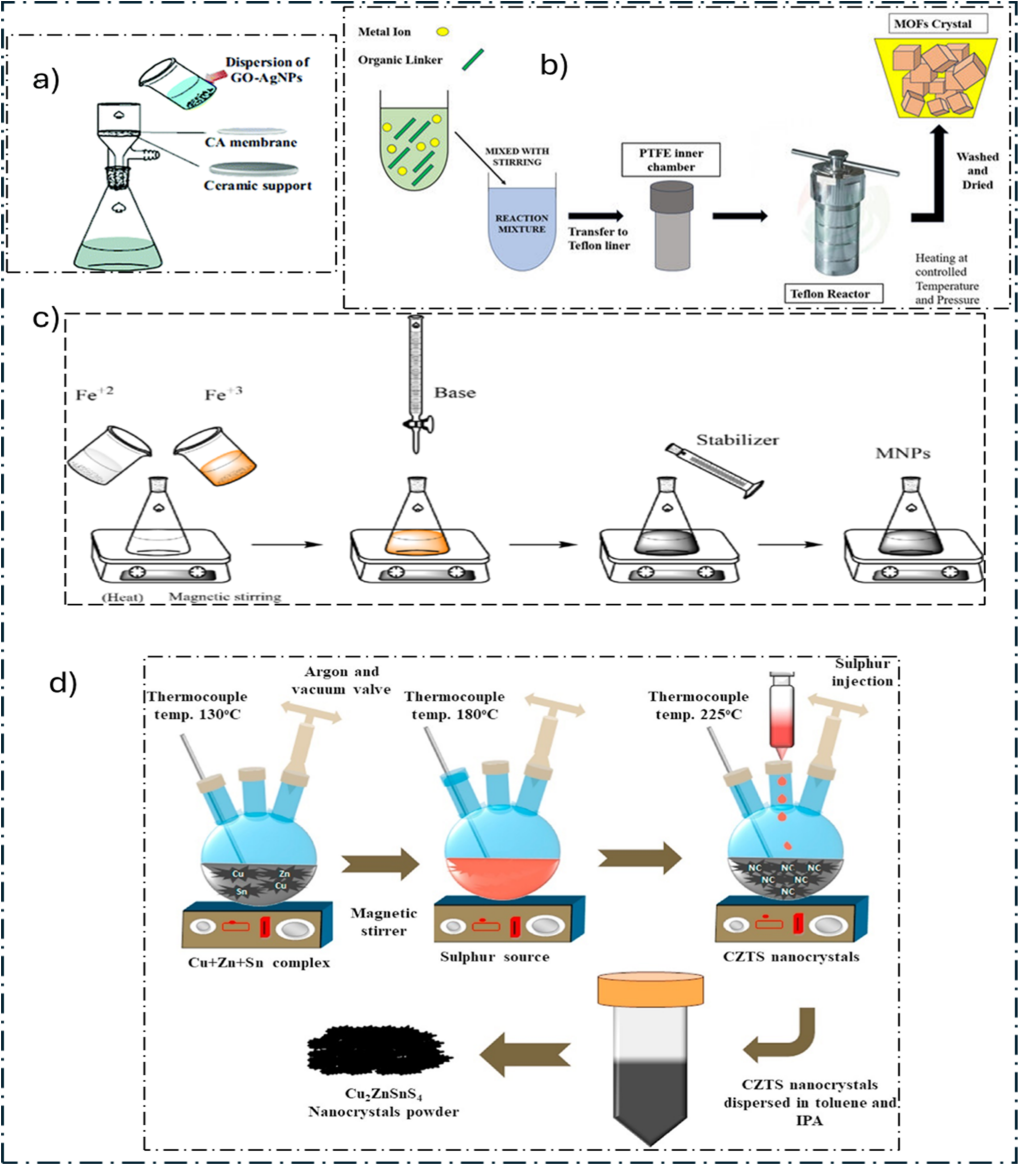
(a) Vacuum filtration method,^82^ (b) hydrothermal/solvothermal technique,^83^ (c) co-precipitation method,^84^ and (d) hot injection method.^85^

This quick and efficient process physically blends different materials to form nanocomposites using vacuum filtering (Fig. 3a). Usually, a mixture of ingredients is made, the filtrate is dried, and the mixture is subsequently filtered using a vacuum. This method makes it simple to change the composition of the blend by altering the weight percentage or concentration of each component.

Vacuum filtration was used by Xu and colleagues^86^ to create a nanocomposite made of graphene, AC, and PPy. During 5000 charge/discharge cycles, the final electrode showed a *C*_sp_ of 178 F g^−1^ at 0.5 mA cm^−2^. It also maintained 64.4% of its *C*_sp_. Meanwhile, Gao and collaborators^87^ used this method to create a graphene/polymer electrode on NF, controlling graphene dispersion by the duration and vacuum pressure.

### 3.7. Hydrothermal/solvothermal technique

The hydrothermal approach (Fig. 3b), which uses superheated aqueous solutions, may be characterized as an ecologically beneficial procedure. It is better than other methods for creating designed nanoparticles that have excellent purity, crystallization, and regulated chemical and physical characteristics because it allows for controlled diffusion in an enclosed environment. It is also an energy-efficient, low-temperature sintering technique, which makes it easy to scale up and apply.^88^ However, this approach makes particle agglomeration less manageable. In the supercritical phase, the dielectric constant and solubility of the solvent vary significantly, leading to increased reaction rates and severe supersaturation, which are conducive to particle creation. When an alternative solvent is used in the place of water, the process is known as solvothermal synthesis. This technique has been used to create a wide range of SC electrodes, such as hexagonal NiCo_2_O_4_ nanostructures,^89^ rod-like hollow CoWO_4_/Co_1−*x*_S,^90^ and CoS_2_-rGO (reduced graphene oxide).^91^

### 3.8. Co-precipitation method

This process (Fig. 3c) provides a simple way to produce sample powder on a large scale. Precipitation occurs when a solute surpasses its solubility limit at a sufficiently high temperature for rapid precipitate formation. However, regulating the shape of the resulting samples is difficult due to the fast precipitation rate. Several supercapacitor structures have been documented by employing this approach. Examples include CoFe_2_O_4_ magnetic nanoparticles synthesized from different precursors^92^ and the Ni_3_(PO_4_)_2_@GO composite.^93^

### 3.9. Hot injection method

In order to properly synthesize monodisperse CdE (S, Se, or Te) quantum dots (QDs), Bawendi and colleagues developed a hot injection approach (Fig. 6d). This technique includes isolating the quick nucleation step from the subsequent development process.^94^ Organometallic reagents were rapidly added to a heated solution containing ligands and surfactant molecules, such as oleic acid, oleylamine, and trioctylphosphine oxide. This enabled immediate nucleation, which led to consistent diffusion-controlled growth in the solution, resulting in smaller QDs forming faster than larger ones. Ostwald ripening resulted from this process, in which larger QDs grew until they reached saturation, whereas smaller ones disintegrated because of their higher chemical potential. The size and form of monodisperse QDs may be effectively controlled by hot injection by varying variables, such as temperature, solvent level, injection velocity, and reaction time.

## 4. Metal oxide-based electrode materials

**Fig. 4:**
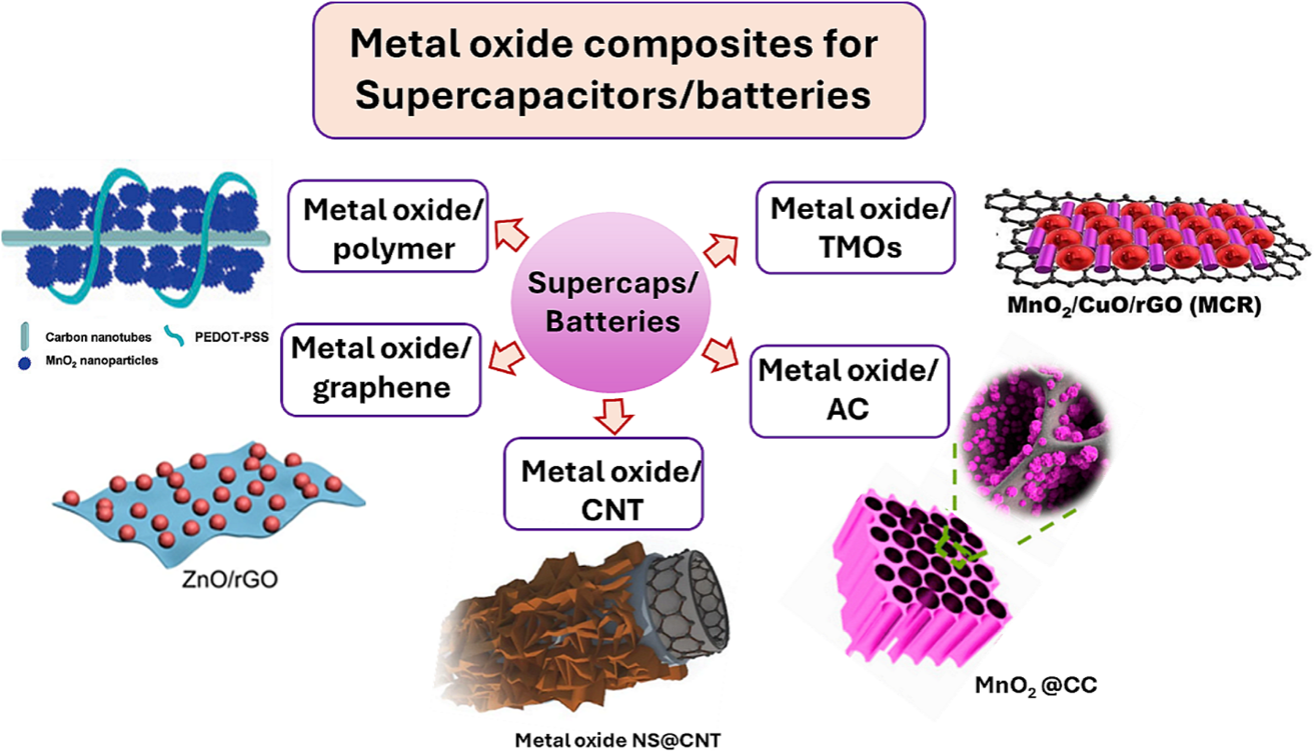
Illustration of several forms of metal composite-based electrode materials for energy storage.^95^

Electrode materials, especially TMO (Fig. 4) composites, have been the focus of significant research on supercapacitor applications. However, these materials have limitations due to poor electron and ion transport and suboptimal conductivity, hindering their electrochemical performance in energy storage. Progress in the transition of metal-based electrode materials is key to surpassing current limits and enhancing battery performance (energy density, specific power, and charge/discharge rates). SC efficiency and practicality are directly improved by these advancements. This review provides a complete summary of recent advancements in electrode materials, particularly those focusing on TMO composites. This study investigates the morphology, composition, and performance of materials, focusing on their applications in hybrid electrode system development. Moreover, this study clarifies approaches to optimize TMO-based hybrid electrode performance, encouraging broader application in energy storage and conversion technologies. This review aims to boost advanced materials development and future SC applications by highlighting challenges and opportunities.

### 4.1. Manganese-based electrode

Because of its noteworthy properties, MnO_2_ has become the most popular pseudocapacitive material for supercapacitors in recent years. These MnO_2_ properties include a high *C*_sp_ of 1370 F g^−1^,^104^ extensive accessibility,^105^ simplicity of processing and ecological friendliness.^106^ Moreover, it is widely known that the crystalline structure of MnO_2_ and its electrochemical characteristics are closely related.^107^Table 4 presents the works of others and their findings with the MnO_2_-based electrode materials.

**Table 4:** MnO_2_-based electrode materials used in other studies

Composite	*C*_d_	*C*_sp_ (F g^−1^)	No. of cycles	Retention rate (%)	*P*_d_ (W kg^−1^)	*E*_d_ (W h kg^−1^)	Ref.
MnO_2_/Fe_3_O_4_/CNTs	1	643	10 000	63	850	52.98	110
MnO_2_/NiCo_2_O_4_	1	1186	3000	65	420	29.6	111
CuO/MnO_2_	8	2690	1500	79	—	—	112
Mn_3_O_4_/MnO_2_	2	181	4000	78	1620	118.3	113
MnO_2_/NiCo_2_O_4_	0.25	634.37	3000	96.3	—	—	114
MnO_2_/CuO	1 mA cm^−2^	261.4 (mF cm^−2^)	1000	90	5040 μW cm^−2^	54.3 μW h cm^−2^	115
MnO_2_/NiMn_*x*_O_*y*_	1	463.5	20 000	94.9	—	—	116
MnO_2_/Cu(OH)_2_	—	283	5000	85	750	18.36	117
MnO_2_/Co_3_O_4_	2	728	11 000	72	1276	64.5	118
ACC@MnO_2_@PEDOT	1 mA cm^−2^	1882.5 mF cm^−2^	10 000	94.6	1.259 mW cm^−2^	0.25 mW h cm^−2^	119
MnO_2_/C, nanosheet	1	258	10 000	93	450	32.6	120

In a study conducted by Zhang and colleagues,^108^ on a 3D spongy NF substrate, they created MnO_2_ layers using the electrodeposition technique. Under 0.6 V deposition potential and 1 A g^−1^ current density (*C*_d_), the material demonstrated an impressive capacitance of 469 F g^−1^. Na_2_SO_4_ was also discovered to have very little solution resistance, and the electrode showed quick response times, which further highlighted its advantageous electrochemical properties.

**Fig. 5:**
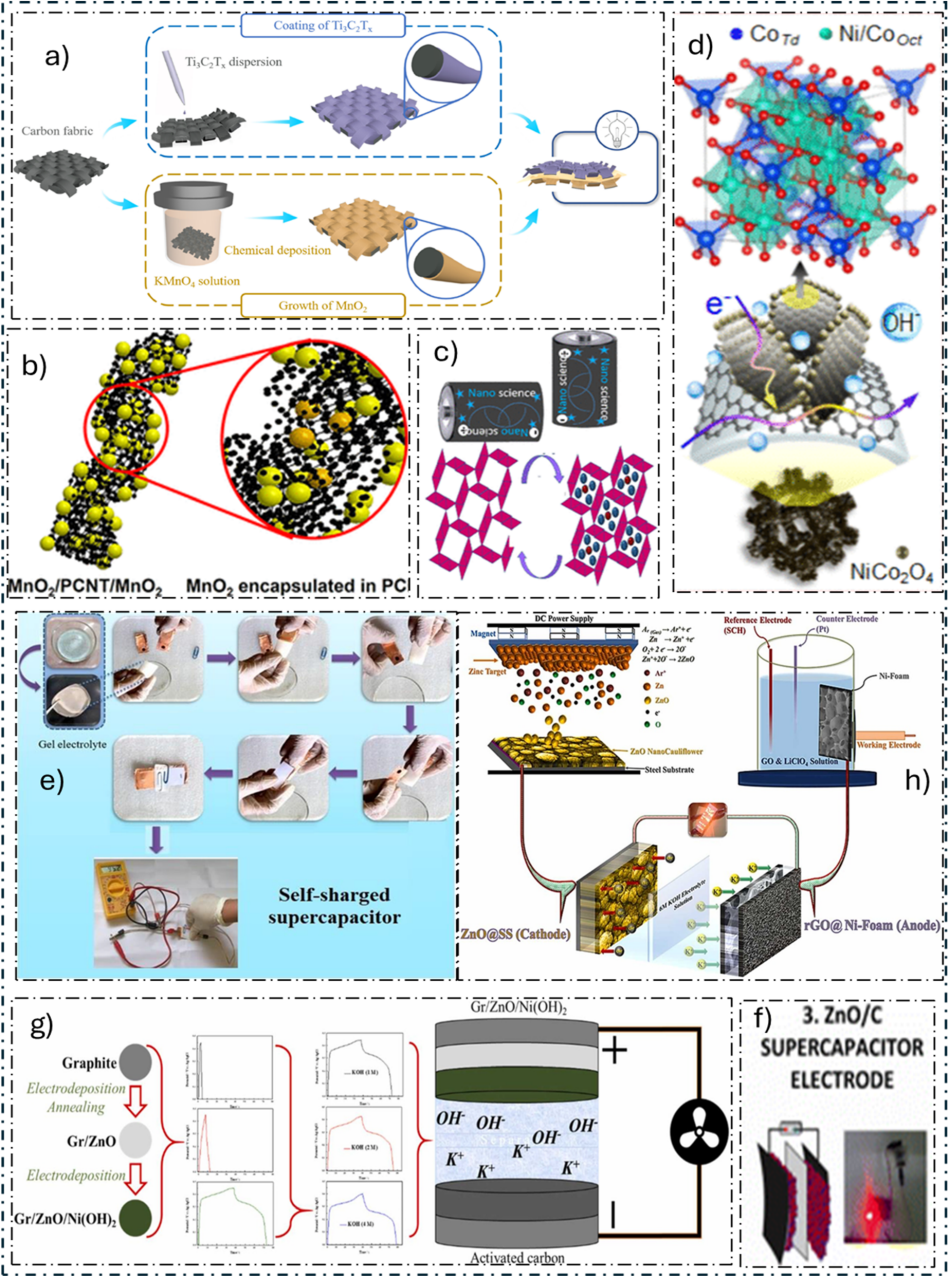
Representative electrode architectures and material systems for advanced supercapacitors. This figure highlights key innovations in electrode design from recent studies: (a) a flexible MnO_2_/MXene electrode for bendable devices. (b) A MnO_2_/porous CNT composite maximizing active sites. (c) Na^+^-preintercalated MnO_2_ nanowires for enhanced conductivity. (d) A 3D NiCo_2_O_4_/rGO nanosheet array on nickel foam. (e) A self-charging device using piezoelectric ZnO. (f) Laser-ablated ZnO/C structures on carbon cloth. (g) A ZnO-coated Ni(OH)_2_ electrode. (h) An asymmetric cell using rGO and ZnO thin films. These examples showcase strategies like nanostructuring, heterostructuring, and hybrid composites to boost performance.^96–103^

Wei *et al.*^96^ (Fig. 5a) developed a unique strategy in which the positive electrode in a neutral electrolyte environment is carbon fabric/MnO_2_ and the negative electrode is MXene/carbon fabric. It was impressive that even after 3000 cycles, it still had an 84% retention rate. Furthermore, even after 1000 bending cycles at angles greater than 90°, two supercapacitors linked in series were able to power an LED for 90 minutes. In a groundbreaking study by Wang *et al.*,^97^ porous carbon nanotubes (PCNTs) were used to create the MnO_2_/PCNT/MnO_2_ (Fig. 5b), a sophisticated supercapacitor nanocomposite (NC). The material demonstrated exceptional cyclic stability, retaining 98% efficiency even after an astounding 6000 cycles of discharge and charge. The copious nanopores found on PCNT walls are responsible for the notable improvement in electrochemical performance. By allowing MnO_2_ nanoparticles (NPs) to penetrate into the nanocavities and onto the PCNT surface, these nanopores created a large number of electroactive sites. Furthermore, the pore shape promoted ion exchange, reducing ion transport distances, while the addition of PCNTs enhanced electrical conductivity. Moreover, the skillful handling of volume fluctuations by PCNTs during charge/discharge cycles contributed to the NC's improved efficiency. Khalid and colleagues^98^ (Fig. 5c) added Na^+^ ions into MnO_2_ nanowires (NWs) before intercalation, acting as a stabilizer and conductivity booster for α-MnO_2_ tunnels. Analyzing the structure, we find that the NWs have a diameter of less than 50 nm and that there are surface fractures that occur after Na^+^ preintercalation, which minimizes dead zones. The oxygen evolution overpotential is increased as a result of Na^+^ preintercalation, according to linear sweep voltammetry studies. This might lead to a wider operating potential window for SCs. In addition to improving conductivity, Na^+^ preintercalation and morphological manipulation lessen the electrolyte's diffusion channel and shield the electrode from pulverization through repeated cycles of charging and discharging. The findings of this study indicate that Na^+^ preintercalation in MnO_2_ can greatly improve electrochemical efficiency, opening the door for the eventual creation of SCs with excellent performance.

Zhou and colleagues^109^ created a conductive NC matrix that uses lighter materials, such as cellulose, in place of heavy metal conductive substrates, including NF, lowering the weight of the device and increasing the SC's utility. Using a straightforward hydrothermal technique, they initially created two distinct architectures of MnO_2_ (MnO_2_@CFCB) on carbon fibers and carbon black. Next, they used cotton fibers and CB conductive slurry to build a self-supporting conductivity-enhanced anode (MnO_2_@CFCBSC). Furthermore, capacitance is further increased by adding a redox-active electrolyte (ammonium iron citrate or AFC) to a neutral Na_2_SO_4_ electrolyte. At a *C*_d_ of 12.1 mA cm^−2^, a 7-layer electrode material arrangement produces 4.9 F cm^−2^ unit area capacitance. This work offers a fresh strategy for advancing ASCs.

### 4.2. Nickel-based electrode

Its superior properties and low cost make nickel oxide a prime electrode material choice in supercapacitor applications. It has outstanding thermal robustness and an amazing theoretical *C*_sp_ of 3750 F g^−1^.^121^ The use of nickel oxide derivatives, such as Ni_2_O_3_, NiO_2_, and NiO, in applications involving energy storage is well known and respected. Their availability in various crystalline forms, including hexagonal, cubic, and monoclinic, increases their adaptability to various energy storage scenarios, particularly for SC applications. To overcome nickel oxide's limited conductivity, a composite made of various materials is used.^122^Table 5 presents other works on nickel-based electrodes.

**Table 5:** Nickel-based electrode materials used in other studies

Composite	*C*_d_	*C*_sp_ (F g^−1^)	No. of cycles	Retention rate (%)	*P*_d_ (W kg^−1^)	*E*_d_ (W h kg^−1^)	Ref.
PPy/NiO	1	679.0	1000	83.9	500.74	94.4	126
PPy/NiO/MWCT	0.5	395.0	5000	90	—	—	127
NiO/N-rGO, wrinkle	1	233	80 000	89	124.9	8.09	128
Ni(OH)_2_@NiCo_2_O_4_, nanosheets	1.25	3250	1000	80	—	—	129
Ni(OH)_2_–MnO_2_@C	2 mA cm^−2^	965.1	5000	93.3	221.4	39.1	130
NiO/C/HS	1	6860.00	5000	83	193	30.5	131
NiO/NiS	2	1063	10 000	93	—	—	132
NiO/MWCNT	5	1200	40 000	∼99	∼140	∼9	133
NiCo_2_O_4_-Co_3_S_4_	1	1468	3000	84.7	400	14.0	134
NiO-Mn_2_O_3_@rGO	1	442	500	91	810	45	125
NiFe_2_O_4_ QDs@Ni-MOF-10	1	852.3 mA h g^−1^	5000	87.3	798	32.5	135

Wei and team^99^ (Fig. 5d) created a 3D structured nanosheet (NS) matrix-like composite (NiCo_2_O_4_/rGO/NF) of reduced graphene oxide, NF, and nickel cobaltite. They used an aqueous coprecipitation–hydrothermal technique with citric acid assistance to build this mixture. A NiCo layered-double-hydroxide precursor is advantageous to the composite because it successfully hybridizes with rGO and provides metal ions with an atomic-level lattice confinement effect. This leads to the surface modification of a rGO-modified NF skeleton with thin NiCo_2_O_4_ NSs (about 113.6 nm × 11.2 nm) made up of NiCo_2_O_4_ (approximately 10.9 nm) vertically staggered. A large surface area, a plentiful mesoporous form, and the exposure of active sites are all provided by this arrangement. Several TMO/graphene/NF composites with remarkable structural durability and efficiency in the storage of energy and associated uses may be designed and built using this synthetic approach. Z. A. Sheikh and colleagues,^123^ studied NW-like porous synthetic MoS_2_/CoNiO_2_. CoNiO_2_ NWs were chosen as the porous backbone because of their edge locations, excellent surface/volume proportion, and outstanding electrochemical properties. In order to produce novel hierarchical 3D porous NW MoS_2_/CoNiO_2_ hybrids with outstanding charge accumulation and effective ion transport capabilities, these NWs were adorned using layered MoS_2_ nanoflakes. This NC offers advantages for preserving charge dynamics, as evidenced by electrochemical measurements that yield a *C*_sp_ of 1340 F g^−1^ at a *C*_d_ of 0.5 A g^−1^.

M. H. BinSabt and colleagues^124^ created graphene oxide (GO) composites in β-Ni(OH)_2_ and β-Ni(OH)_2_/graphene foil electrodes using electrochemical cyclic voltammetry with a 0.5 M KOH solution. The hybrid material's *C*_sp_ was greatly increased by the addition of graphene oxide layers. In particular, with the introduction of four layers of GO, the *C*_sp_ values increased from 110 F g^−1^ to 280 F g^−1^. Furthermore, even after 500 cycles of discharging and charging, the SC showed remarkable stability, with its capacitance values remaining almost unaltered. D. Bejjanki and collaborators^125^ established a quick co-precipitation technique for rGO, manganese oxide, and nickel oxide to form a ternary composite that may be used as an electrode material. The results demonstrated how well rGO may be added to TMOs to improve electrode efficiency. As a result, the synthesis approach shows the potential for scalable procedures in the production of SC electrode materials. Furthermore, across 500 cycles of charging and discharging at a *C*_d_ of 1 A g^−1^, the ternary composite surpassed its binary opposition, demonstrating greater reliability and a capacitance retention rate of 91%. This improvement was attributed to the synergistic interactions between rGO and NiO-Mn_2_O_3_, which strengthened conductivity, ionic transport, and faradaic reactions. As a result, an appealing electrode material for SC uses is the NiO-Mn_2_O_3_@rGO combination. S. Verma and colleagues^100^ produced a self-charging ASC with piezoelectric characteristics using NPs of ZnO as a piezoelectric substance and combining them into an ASC (Fig. 5e). Ni and Mg-Co NW electrodes are binder-free in this novel device. Furthermore, a ZnO-based separator operates as an energy harvester when combined with a KOH electrolyte. The electrochemical measurements show remarkable cyclic stability; over a total of 10 000 galvanostatic charge–discharge cycles, the Mg-Co and Ni NW electrodes showed capacitive retention levels of 100% and 96.5%, respectively. In addition, the gadget produces an ultimate voltage of about 99 mV when compressed by pressing the thumb. This methodology provides a simple and economical technique to create a self-charging powerhouse SC, opening the door for the creation of the next wave of integrated energy storage and harvesting devices.

### 4.3. Zinc-based electrodes

The applications of zinc oxide (ZnO) are extensive, ranging from sensors and optoelectronics to energy storage, spintronics, and catalysis. What makes it attractive is its superior electrochemical properties, cost-effectiveness, and sustainability. ZnO composites mixed with AC, graphene, and CNTs are particularly interesting electrode materials for SC applications due to extensive research.^154^Table 6 presents the ZnO-based electrode material from other studies.

**Table 6:** ZnO-based electrode materials used in other studies

Composite	*C*_d_	*C*_sp_ (F g^−1^)	No. of cycles	Retention rate (%)	*P*_d_ (W kg^−1^)	*E*_d_ (W h kg^−1^)	Ref.
Al_2_O_3_-ZnO composite	1	463.7	5000	96.9	1360.9	10.3	138
ZnO-CoO@NC microsphere	2	154	40 000	92	5634.5	5.5	139
NiO–ZnO@g-C_3_N_4_	0.5	726.7	1000	78	160	7.91	140
ZnO@Mo–C composite	1	900	6000	97	992	30	141
ZnO-SnO_*x*_ composite	0.5	810.89	5000	119	374	50	142
ZnO/CNT	—	189	1000	96	2250	10.7	143
PANI-RGO-ZnO	0.05	40	5000	86	403	5.61	144
ZnO-CoSe_2_	1	450.7	5000	105.6	825	22.35	145
PPy/ZnO	0.5	161.02	5000	70.71	5980	4.62	146
ZnO@MOF@PANI	1	340.7	5000	82.5	—	—	147

S. E. Berrabah and colleagues^102^ created a powerful SC electrode by improving a binder-free Ni(OH)_2_ electrode with a thin coating of ZnO (Fig. 5g). Two steps were taken in the synthesis procedure, which was carried out on a graphite substrate. First, graphite was electrodeposited with a layer of ZnO (Gr/ZnO). This layer supported the subsequent electrodeposition of Ni(OH)_2_. Gr/ZnO, Gr/Ni(OH)_2_, and Gr/ZnO/Ni(OH)_2_ were compared, and the results showed that ZnO/Ni(OH)_2_ had a positive synergistic impact on energy storage capacity. Furthermore, with a *P*_d_ of 0.75 mW cm^−2^, the HSC made of Gr/ZnO/Ni(OH)_2_//AC produced an impressive areal *E*_d_ of 65.1 μWh cm^−2^. N. N. Tarasenka and colleagues^101^ developed a novel two-step process to create ZnO/C NCs in liquid media (Fig. 5f). The first stage involved pre-treating a carbon cloth substrate with DC glow discharge plasma, and the second step used laser ablation aided by an electric field. Concurrently, these composites were arranged on the pre-treated carbon fabric into ordered architectures, resulting in an SC electrode. The treated carbon cloth functioned as the cathode in the electrical circuit throughout the production of these structures, while a zinc target served as the anode through the laser ablation of zinc in water. It was discovered that the content of the liquid medium and the direction of the applied electric field affected the properties of the resultant nanostructures. The produced nanomaterial's nanoflower-like form has been demonstrated by scanning electron microscopy (SEM) research, suggesting a sizable surface area suitable for SC uses. These results highlight the produced ZnO/C NC's excellent energy storage potential as a SC material.

A. Kumar and collaborators^103^ (Fig. 5h) created an ASC using rGO@Ni and ZnO@stainless steel (SS) as electrodes. Reactive DC magnetron sputtering was used to create the ZnO thin film electrode, and electrodeposition was used to place the rGO electrode. The electrochemical characteristics of the thin film electrodes were assessed in an aqueous KOH electrolyte solution with a concentration of 6 M. rGO@Ni//ZnO@SS, the unique ASC, performed better in terms of capacitance than conventional metal oxide materials. These great electrochemical characteristics were proven by the ASCs. The superior efficiency of these nanostructured ASCs offers great promise for use in handheld devices and electric cars.

In their study, I. Shaheen and collaborators^136^ created an HASC electrode using an affordable and efficient electrophoretic deposition technique. They used a sol–gel synthesis method to create ZnO/CuO and ZnO/CuO/rGO heterostructures; then, they used electrophoretic deposition to deposit thin, homogeneous layers of ITO onto the substrate by applying 1 V for 20 minutes at a scan rate of 50 mV s^−1^. On the other hand, the ZnO/CuO/rGO heterostructure showed increased *C*_sp_, measuring 1235 F g^−1^ at 5 A g^−1^ and 2305 F g^−1^ at 2 mV s^−1^. The ZnO/CuO/rGO heterostructure showed noteworthy promise for real-world application, as it obtained the maximum specific *E*_d_ of 110 W h kg^−1^. This paper provides an outline for the ecological, economical, and productive manufacturing of flexible electrodes, which might be used in energy storage on a large scale. V. Shanmugapriya and colleagues^137^ introduced combined ZnO/SnO_2_:rGO (ZSR) and ZnO/SnO_2_ mixed metal oxides (ZS) as two types of NCs. They were able to validate the orthorhombic structure of SnO_2_ in ZSR NCs and the hexagonal structure of ZnO in ZS NCs using an XRD technique. The electrochemical characteristics of the ZS and ZSR NCs were assessed by employing redox additive electrolytes (RAEs) and 1 M Na_2_SO_4_. The working electrodes changed by the ZSR NCs demonstrated an impressive *C*_sp_ of 3238 F g^−1^ in the RAE. Additionally, after 5000 cycles, the electrochemical capacitance retention was 91.54%. Similarly, the greatest *C*_sp_ of 89.19 F g^−1^ was demonstrated by ASC based on manufactured ZSR NCs. After 5000 cycles under RAE, the ZSR NC-based ASC showed a better electrochemical preservation of 94.01%. These results suggest that ZSR NCs and that the additional Na_2_SO_4_ 0.1 M K_4_[Fe(CN)_6_] redox enhancers could serve as potential electrode and electrolyte materials for enhanced SCs.

### 4.4. Cobalt-based electrodes

Cobalt oxides have attracted significant interest across various research fields owing to their straightforward synthesis methods, abundance in the Earth's crust, non-toxic nature, cost-effectiveness, and environmental friendliness. These attributes have positioned them as focal points in numerous scientific studies and applications, particularly in the field of SCs. There are several types of cobalt oxides, including CoO,^152^ Co_2_O_3_,^153^ CoO_2_,^154^ and Co_3_O_4_.^155^ The most widely used of these are Co_3_O_4_ and CoO because of their extraordinary physical and chemical properties as well as their amazing heat stability. Table 7 presents the cobalt-based electrode materials from other research.

**Table 7:** Cobalt-based electrode materials used in other studies

Composite	*C*_d_	*C*_sp_ (F g^−1^)	No. of cycles	Retention rate (%)	*P*_d_ (W kg^−1^)	*E*_d_ (W h kg^−1^)	Ref.
Co_3_O_4_-rGO	1	636	1000	95	225	35.7	161
NPC-Co_3_O_4_	2.25	885	10 000	94	—	—	162
Co_3_O_4_/CNF	1	80	5000	94	0.9	∼10	163
Co_3_O_4_/NCFY	—	713	8000	92	209	45.5	164
Co_3_O_4_ NGC	2	128.43	5000	92.1	399.9	45.66	165
Co_3_O_4_-NiO/GO	1	883	3000	82	825	50.2	166
Co-Co_3_O_4_@CNT-NC	1	823.4	10 000	93.6	1601.1	46.7	167
Co_3_O_4_/graphene	—	140	1000	95	856	2.38	168
MWCNT_*x*_@Co_3_O_4_	1	206.89	1000	87.2	800	17.78	169
CuCo_2_O_4_/CuO	1	458	8000	90	—	—	170
FCO-31	1	2834	1000	78	600	6.5	171

#### 4.4.1. Co_3_O_4_

Liu and colleagues^156^ created a P-doped CNT@MnCo_2_O_4_/Co_3_O_4_ layer. By adding P heteroatoms, MnCo_2_O_4_/Co_3_O_4_'s electronic configuration is successfully adjusted, improving electrical conductivity and encouraging the Faraday response. The results of electrochemical testing indicated that phosphorus doping significantly improved the capacity and efficiency of CNT@MnCo_2_O_4_/Co_3_O_4_. The *C*_sp_ of the P-CNT@MnCo_2_O_4_/Co_3_O_4_ electrode was 1064.4 F g^−1^. This work demonstrates the potential of phosphorus-doped pseudocapacitive materials in advanced charge storage using a novel synthesis method for efficient hybrid energy storage.

**Fig. 6:**
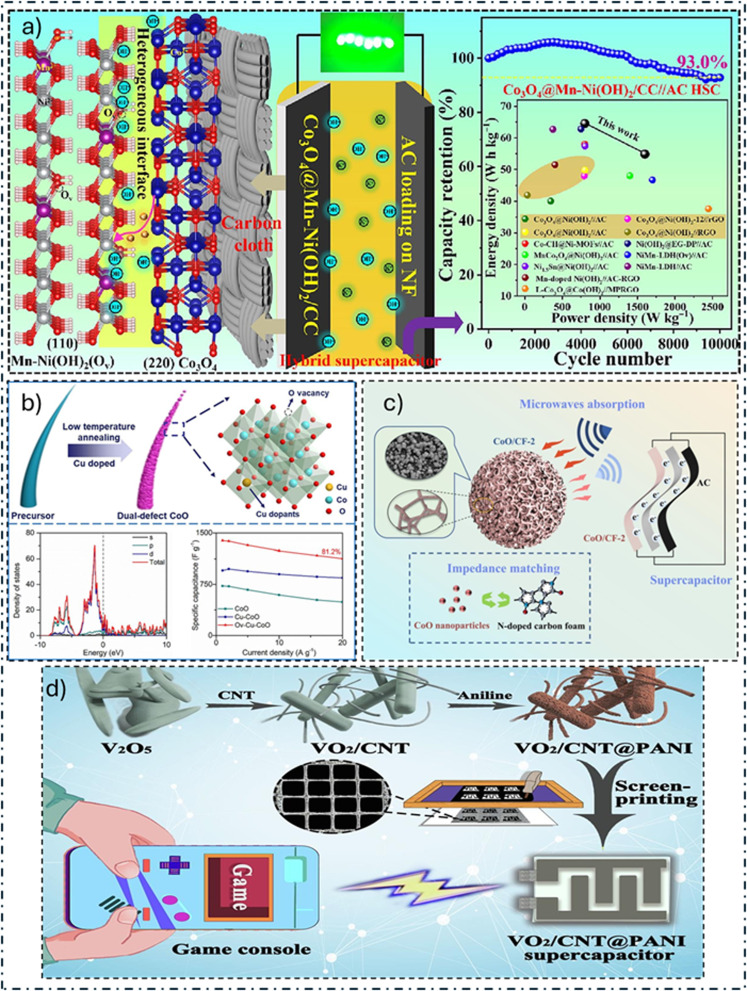
Advanced design strategies for high-performance cobalt-based supercapacitor electrodes. This figure showcases key structural innovations: (a) a Co_3_O_4_@Mn-Ni(OH)_2_ core–shell heterostructure for enhanced stability, (b) multi-defect engineered Ov–Cu–CoO to boost redox activity, (c) a 0D/3D CoO/N-doped carbon foam composite to prevent aggregation, and (d) a VO_2_/CNT@PANI core–shell design (included for its generalizable coating principle) to improve cycling life. These architectures aim to maximize conductivity, active site exposure, and long-term durability.^148–151^

Wang and colleagues^148^ (Fig. 6a) created a core–shell heterostructure known as Co_3_O_4_@Mn-Ni(OH)_2_/CC by depositing oxygen-vacancy-containing Mn-doped Ni(OH)_2_ NSs atop Co_3_O_4_ NRs embedded in carbon cloth (CC). The above results outperformed the other designs of HASC, such as Mn-Ni(OH)_2_/CC//AC HSC (53.9%) and Co_3_O_4_@Ni(OH)_2_/CC//AC HASC (36.5%). The electrode's long-term stable cycling was found to be influenced by the addition of Mn and the consequent oxygen vacancies, which improved conductivity and hindered permanent phase shifts of Ni(OH)_2_ during charging and discharging steps, according to analysis and density functional theory (DFT) computations. These results highlight how well heterostructure design and sensible doping may improve the efficiency of the materials used for electrodes in SCs.

S. M. Qashqay and collaborators^157^ presented a unique remedy in the shape of multilayer Co_3_O_4_/VS_4_/rGO-SDBS@NF electrodes that are self-supporting and without binders. A straightforward two-step hydrothermal procedure was used to make these electrodes. Growing on VS_4_/rGO-SDBS flower-shaped NS arrangements on NF surfaces, the Co_3_O_4_ NPs provide sufficient space dispersion for growth and shrinkage during cycles of charging and discharging. Furthermore, over 5000 cycles, the Co_3_O_4_/VS_4_/rGO-SDBS@NF electrode retains 97.4% of the original capacitance, demonstrating exceptional stability during cycling. These promising results highlight the binder-free Co_3_O_4_/VS_4_/rGO-SDBS@NF electrode's potential as a high-performing SC electrode in the not-too-distant future. This study presents novel techniques for producing highly efficient SC electrodes that do not require binders. Li and colleagues^158^ have effectively created a composite known as Co_3_O_4_@CNTs@NF by joining an oxygen vacancy-rich Co_3_O_4_ NW array with CNTs on NF. Inside the Co_3_O_4_@CNT composite, the addition of CNTs and oxygen vacancies enhances the conductivity of electricity, expands the pool of available active sites, and speeds up faradaic redox processes. Co_3_O_4_@CNTs and NF form a strong connection that yields exceptional capacitive characteristics and exceptional cyclic endurance. In an ASC, this electrode may act as the anode and AC@NF may act as the cathode. At 273.4 W kg^−1^, the Co_3_O_4_@CNTs@NF//AC@NF SC that is produced has a high *E*_d_ of 57.6 W h kg^−1^. This work provides insightful information on the development of materials for electrodes for the upcoming SC era.

#### 4.4.2. CoO

The precise formation of Co_2_V_2_O_7_ with various topologies atop 3D urchin-like CoO microspheres was accomplished by Jiao and colleagues.^159^ They succeeded in creating Co_2_V_2_O_7_ with various structures, such as 3D hollow nanocages, 3D nanocages connected to polygonal nanosheets, 2D honeycomb-like NSs, and 2D irregular NSs on CoO, by precisely adjusting the vanadium concentration. Optimizing Co_2_V_2_O_7_'s electrochemical activity was made possible by modifying both its structure and composition. Most remarkably, the refined cobalt vanadate with polygonal NSs backed by a CoO skeleton and hollow cages connecting to them showed remarkable energy storage capability. Additionally, an ASC fabricated from this material exhibited an exceptional capacitance preservation of 84.65% over 5000 cycles. In conclusion, the suggested strategy of using CoO-supported structure-controllable cobalt vanadate shows great potential for the creation of supercapacitor electrode materials.

Y. Feng and collaborators^149^ (Fig. 6b) effectively introduced Cu-CoO NWs (Ov–Cu–CoO) and oxygen vacancies as part of a multi-defect technique. This strategy efficiently modifies the electronic makeup and distribution of charges by utilizing the dual defect synergistic effect, which boosts redox chemistry and electrical conductivity. This work presents a practical method for improving CoO's electrochemical properties that can be easily extended to additional TMOs. Z. Jiao and colleagues^160^ presented an innovative multi-step approach for creating a 3D/3D composite construction, whereby 3D sea urchin-like CoO microspheres are attached onto 3D hollow NiCo LDH nanocages. The results show that the hollow NiCo LDH provides a sufficient number of redox-active sites, and the 3D CoO framework functions as an effective and long-lasting channel for ion transport. Computational DFT studies indicate that the CoO@NiCo LDH heterostructure possesses superior OH− adsorption and a high density of states close to the Fermi level, thus implying better electrical conductivity and electrochemical reaction dynamics. Moreover, at 39.54 mW cm^−3^, the AHSC built with this material has an Ed of 5.59 mW h cm^−3^. These results highlight how well the 3D/3D architectural recombination technique functions in the design of electrode materials and provide new opportunities for the creation of additional energy-related materials. H. Chen and collaborators^150^ carbonized a melamine foam to create an *N*-doped 3D carbon foam (CF) (Fig. 6c). The authors next used a solvothermal technique to combine CoO NPs with 3D CF to form a 0D/3D architecture. In addition to acting as a conductive structure and growth substrate, CF also improves electrical conductivity across the different parts and resolves problems associated with the aggregation of CoO NPs. CoO/CF has better SC efficiency than each of the components, with a remarkable *C*_sp_ of 221 F g^−1^ at 1 A g^−1^, which is achieved by the combined influence of double-layer capacitance and pseudocapacitance.

### 4.5. Vanadium-based electrodes

Five valence electrons reside in vanadium's outermost shell, making it multivalent. It may be found in oxidation states like +5, +4, +3, and +2. The exceptional pseudocapacitance capability of this system is attributed to its diverse states. Vanadium-based materials are among the best candidates for high-energy electrochemical capacitors because of their exceptional *C*_sp_, strong electrical conductivity, excellent electrochemical reversibility, and extended cycle life. Vanadium compounds have notable stability in the +5 oxidation state, but the +4 and +2 states exhibit less stability. Because of their important qualities and uses, VO_*x*_, VN, and vanadium bronzes are among the well-studied vanadium complexes.^172–174^Table 8 presents other studies on vanadium-based SCs.

**Table 8:** Vanadium-based electrode materials used in other studies

Composite	*C*_d_	*C*_sp_ (F g^−1^)	No. of cycles	Retention rate (%)	*P*_d_ (W kg^−1^)	*E*_d_ (W h kg^−1^)	Ref.
NiO/V_2_O_5_/rGO	—	1265	4000	92	553	41.4	178
V_2_O_5_-CS	5	612	1500	58	—	—	179
AC//CeVO_4_/PPy	—	116	10 000	92	676	52.2	180
VO_*x*_@C core–shell nanorod	5	437	2000	88.3	2136	8.9	181
V_2_O_5_@CFC-30	5	57	10 000	94	2728	17.7	182
V_3_O_7_-rGO-PANi	0.2	579	2500	94	—	—	183
ZnV_2_O_6_@PPy	1	723.6	3000	93	748.7	34	184
V_2_O_5_@PPy	0.5	307	1000	82	161	37	185
GO/V_2_O_5_/polyaniline(GVP)	1	273	13 000	61	1636	54.6	186
VO_2_@S-rGO-1	0.5	204.2	10 000	75	426.0	32.6	187

Despite its promising theoretical capacitance, addressing challenges such as inferior cycling life and lower *E*_d_ remains crucial. To tackle these issues, C. Chen and collaborators^151^ (Fig. 6d) devised a method to prepare VO_2_ NRs wrapped with CNT, followed by polymerization of a PANI shell. Owing to the expanded conductivity of CNT and the stabilizing effect of the PANI shell, the end product VO_2_/CNT@PANI composite exhibits a high *C*_sp_ and outstanding stability during cycling of around 88.2% in excess of 5000 cycles. Using the exceptional rheological characteristics of the prepared inks, they created an in-planar VO_2_/CNT@PANI SSC with a well-organized structure. With a *P*_d_ of 387.5 μW cm^−2^, this device achieves an outstanding areal *E*_d_ of 99.57 μW h cm^−2^ and, even after extended usage, retains around 87.6% of its starting capacitance. They also used two SSCs connected in series to power a portable gaming console for more than 120 seconds. Therefore, this work offers a general approach that uses coating and combination methods to improve the electrochemical characteristics of powerful flexible SCs. Using a by-product generated during the production of PANI 54.69%: ZnO 7.81%: VO_2_ 37.50% (PZnV) NC, A. Viswanathan and colleagues^175^ suggested an environmentally friendly energy storage approach. This environmentally friendly technology shows a 23% improvement in energy storage over traditional techniques using 1 M H_2_SO_4_. When the acidified by-product is present, the PZnV NC exhibits enhanced energy storage properties. PZnV is noteworthy for its distinct feature, which increases energy storage with the number of cyclic voltammetry cycles in a solution of 1 M H_2_SO_4_. After 12 312 cycles, the PZnV reaches its maximum storage capacity with *C*_s_ of 440.5 F g^−1^. Furthermore, at 0.4 V s^−1^, the PZnV exhibits high stability of up to 16 812 cycles. B. M. Ndiaye and co-authors^176^ employed an environmentally friendly approach to synthesize composites based on VO_2_ (VO_2_@C/SO_4_^2−^) and Ni-VO_2_ (Ni-VO_2_@C/SO_4_^2−^). An SC built with AC serving as the anode and the Ni-VO_2_@C/SO_4_^2−^ composite serving as the cathode were used in order to evaluate practical applicability. The remarkable electrochemical efficiency is ascribed to the combined influence of Ni doping and the additive effects of VO_2_, SO_4_^2−^, and AC. These results validate the Ni-VO_2_@C/SO_4_^2−^ composite's potential application in creating sophisticated cathodes for the next SC era.

R. Thangappan and colleagues^177^ manufactured VO_2_/rGO nanowhisker composites by applying a simple hydrothermal process. VO_2_ and 2D flexible graphene sheets were made throughout the construction of the VO_2_/rGO architecture, leading to the creation of an interconnected porous microstructure aided by hydrogen bonding. Efficient recharging and ion transport inside the electrode are made possible by this arrangement. Because of the VO_2_ nanowhiskers' pseudo-capacity contributions and its hierarchical network topology, the composite electrode performs very well electrochemically. The composite electrode was used in a 1 M Na_2_SO_4_ solution for electrochemical studies. An excellent cycle life was demonstrated by the specific capacitance, which preserved 96.3% through 5000 continuous discharge cycles at 0.6 A g^−1^. Furthermore, the substance demonstrated a respectable high energy density and a high Pd, with an *E*_d_ of 48.07 W h kg^−1^ at a *P*_d_ of 213.6 W kg^−1^. The impressive results stem from the synergistic effects of VO_2_ and graphene, specifically VO_2_'s superior redox activity and graphene's high electrical conductivity.

### 4.6. Ruthenium-based electrodes

RuO_2_ exhibits many significant characteristics that make it an excellent choice for use in SC. These characteristics include large potential windows, quick charge/discharge speeds, higher electrochemical reversibility and highly reversible redox reactions, remarkable thermal stability, excellent conductivity, and an impressive *C*_sp_, which can endure multiple cycles. RuO_2_'s redox activity is particularly important since it is essential for electrochemical energy storage devices. RuO_2_ nanomaterials also exhibit remarkable properties, including a large theoretical *C*_sp_ that ranges from 1400 to 2000 F g^−1^.^188^Table 9 contains research works based on ruthenium-based electrode materials.

**Table 9:** Ruthenium-based electrode materials used in other studies

Composite	*C*_d_	*C*_sp_ (F g^−1^)	No. of cycles	Retention rate (%)	*P*_d_ (W kg^−1^)	*E*_d_ (W h kg^−1^)	Ref.
RuO_2_/rGO quantum dots	1	1120	10 000	89	—	—	193
GO/MWCNT/RuO_2_	0.5	514.9	5000	94.38	8033	37.96	194
MWCNT/ruthenium hydroxide aerogels	0.5	420.3	5000	96.38	8360	36.6	195
MWCNT-PTh-Ru/Pd	0.3	86.0	—	—	280.43	10.75	196
rGO@Ru:V_2_O_5_	1.25	1185	2000	94	570	20.92	197
RuNi_2_O_4_/rGO	1	792	10 000	93	500	110	198
RuO_2_@Ru/HCs	0.5	318.5	5000	92	100	6.76	190
ROKF-2	1	121	5000	90	500	113	199
Co_3_O_4_@RuO_2_/NGO	0.5	472	5000	97	—	—	200
RM NPs@RGO	—	641	1000	∼100	167	23	201
RuO_2_@S + T	—	1865.7	8000	93.9	9200	10.2	202

Chandrashekhar R. and collaborators^189^ observed significant alterations in both the performance and morphology of electrochemical supercapacitors through Co doping. According to their work, 1.00 mol% Co-doped RuO_2_ has potential uses in supercapacitors. Improved electrical conductivity and a more porous shape are the reasons for the increase in *C*_sp_. This study shows that atomic doping, achieved *via* chemical spray pyrolysis, is a useful strategy for improving RuO_2_'s electrochemical supercapacitive efficiency. Zhao and colleagues^190^ developed a cost-effective electrode material utilizing hollow carbon (HC) to make RuO_2_@Ru/HCs using a hydrothermal technique. Using this technique, a structure with a RuO_2_ core encircled by Ru NPs encapsulated in a carbon shell was produced. The carbon layer's inclusion of Ru NPs, carefully positioned, maximized conductivity and active sites for improved pseudocapacitance. The material's ability to store energy was further enhanced in the presence of the RuO_2_ core. The material exhibited remarkable performance after optimizing the ruthenium content to 0.92% by mass. These successes are explained by the material's wide pore size distribution, making it easier for protons and electrons to travel. An SSC that could effectively light an LED bulb was constructed by employing this material. RuO_2_ was added to wood carbon by Zhang and colleagues^191^ to create a unique electrode material that can support itself. This substance showed outstanding morphological stability. Remarkably stable, SPWC15-800 demonstrated a capacitance preservation of 91.25% after a total of 12 450 cycles. The comparable *E*_d_ in the two-electrode assessment at 0.1 A g^−1^ was 3.765 W h kg^−1^, demonstrating the potential for self-supporting materials using wood and carbon.

A new composite called 1D-RuO_2_–N-doped carbon (1D-RuO_2_/C) was presented by B. K. Mana and associates.^192^ It was created using a straightforward thermal process and intended for use in supercapacitors. The 1D-RuO_2_/C composite's superior electrochemical performance stems from its high surface area, the synergistic interplay between RuO_2_ and the carbon support, and the unique one-dimensional structure of RuO_2_. The 1D-RuO_2_/C composite, according to scientists, has interesting uses in energy storage technology.

## 5. Metal sulfide-based electrode materials

**Fig. 7:**
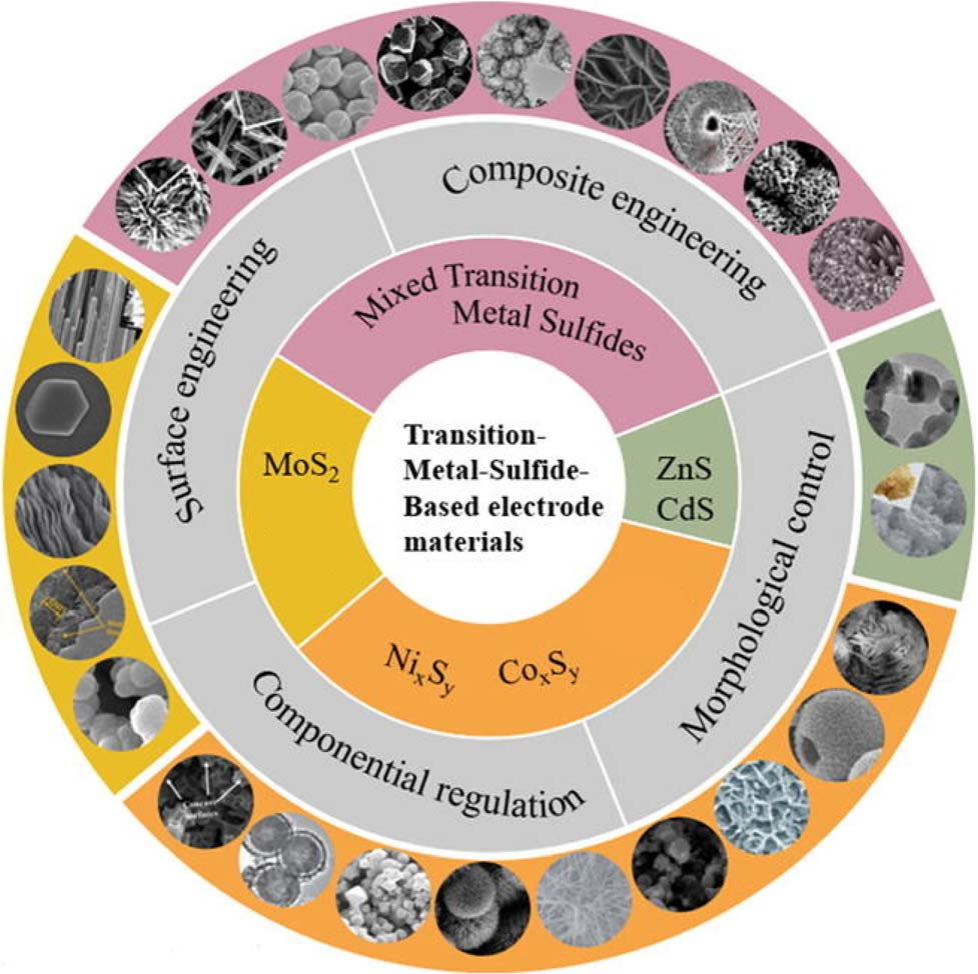
Graphical depiction of various types of metal sulfide composite-based electrode materials for energy storage applications.^203^

Transition metal sulfides (TMSs) (Fig. 7) have many advantages compared to other TMEMs. These advantages include higher storage capacity, better electrical conductivity, excellent redox properties, increased specific capacitance, faster electron/ion diffusion, and improved reversibility. As a result, TMSs have a longer life cycle. Table 10 shows recent studies using TMS for SC material.

**Table 10:** Parameters of various metal-sulfide-based electrode materials

SC	Potential window in volts	Fabrication technique	*E*_d_ (W h kg^−1^)	*C*_sp_ (F g^−1^)	*P*_d_ (W kg^−1^)	Ref.
CoS//AC	1.8	Solvothermal decomposition	5.3	47	18 000	227
Ni_3_S_2_//CoNi_2_S_4_	1.0	Hydrothermal	6.6	54.92	820	228
ZnCoS//P rG	1.5	Chemical precipitation + ion exchange	17.7	90	435	229
MnCo_2_S_4_//rGO	1.6	Hydrothermal	31.3	2067	800	230
NiCoS@PPy//AC	1.6	Template	34.4	97	799	231
Ni/Co-MOF//AC	1.6	Etching	36.9	103.9	1066.4	232
MnS//AC	1.60	Decomposition	37.6	110.4	181.2	233
NiMoS/CNT//AC	1.6	Hydrothermal	40	108	400	234
NiCuCoS_4_//AC	1.65	Hydrothermal	40	105.8	412.5	235
β-NiS@Ni//AC	1.5	Hydrothermal	40	136	720	236
CuCo_2_S_4_//N-DLCHs	1.6	Solvothermal	40.2	113	799.1	237
NiCoS//AC	1.8	Sulfuration	41.4	92	414	238
Ni-Co-S/G//PCNS	1.6	*In situ* chemical conversion	43.3	122	800	239
CoNiS//AC	1.5	Hydrothermal	50	160	783	240
NiS_2_//rGO	1.4	Hydrothermal	50.35	184.9	2260	241
MoS_2_/NiCo_2_S_4_@C HMSs//AC	1.6	Self-template	53.01	120	4200	242
NiCo_2_S_4_/C//AC	1.5	Solvothermal	60.2	192	375	243
NiCo_2_S_4_@rGO//G SWCNHS	1.6	Solvothermal	60.9	171.3	1400	244
NiCoMnS_4_//AC	1.7	Hydrothermal	68.2	170.1	850.1	245
NiCo_2_S_4_/MXene//AC	1.7	Electrostatic assembly	68.7	171.2	850	246
Ni_2_S_2_@PEDOT//aqueous//AC	1.6	Hydrothermal	85.6	243.6	400	247
CoCuMnS//AC	1.6	Sonochemical	88.7	375	820	248
CoMnS//AC	1.6	Electrodeposition	106	118	4000	249
ACF//NTO/ACF	3.0	Hydrothermal	127.73	76.8	95.8	250
Zn-S/RGO/PEDOT symmetric	1.6	Hydrothermal	349.7	722.0	18 000	251

### 5.1. Manganese-based electrodes

Manganese sulfide (MnS) is a particularly good option among the many TMSs for SC electrode materials. This is explained by its low redox potential, plenty of supplies, inexpensive cost, and eco-friendliness.^204^ Additionally, an electrode material's ability to store energy is greatly influenced by its shape and crystal arrangement.

A research team led by A. M. Zardkhoshoui^205^ created hollow α-MnS@Co_3_S_4_ spheres (NSH-MCS) built from nanosheets with a distinctive shape that function as incredibly efficient electrodes. NSH-MCS is formed as a consequence of the Mn-G@ZIF-67 being subsequently sulfidated. This unique shape inhibits the formation of nanosheets while providing an abundance of mass and electron transport pathways. Furthermore, the NSH-MCS electrode shows exceptional performance when incorporated into an HSC with an AC negative electrode. This study's simple methodology opens the door to the effective creation of a wide range of TMSs for various uses.

J. Gao and colleagues^206^ developed a unique method using template and phase-controlled techniques to create carbonaceous frameworks that resemble bird's nests and have hybrid phases of α-MnS and γ-MnS (α/γ-MnS@CFs), especially for SCs. This special structure with both MnS phases resolves volume variations during the cycle, improves electrical conductivity, and guarantees high *C*_sp_ and remarkable stability. During 5000 cycles at 10 A g^−1^, the α/γ-MnS@CFs electrode retains 84.1% of its capacity, indicating exceptional performance. Furthermore, outstanding findings are obtained when integrated into an HSC device, such as an *E*_d_ of 65.2 W h kg^−1^ at 953.5 W kg^−1^ and extraordinary stability during cycling with 69.8% preservation over 5000 cycles at 10 A g^−1^. With its potential uses in energy storage systems, this study offers a novel method for producing TMSs with polycrystalline properties. M. M. Momeni and co-authors^207^ employed a straightforward electrodeposition technique to fabricate flower-like MnS@V_2_O_5_-BiVO_4_. Sample S1, the ideal MnS@V_2_O_5_-BiVO_4_ electrode, demonstrated an impressive *C*_sp_ of 41.6 F g^−1^ at a *C*_d_ of 10 μA cm^−2^, which was 7.5 times more than the capacitance of the naked V_2_O_5_-BiVO_4_ electrodes. After one cycle, this sample showed a considerably increased photocurrent density of 115 μA cm^−2^, which is 3.3 times greater than that of the naked sample and indicates enhanced electron–hole separation. This is due to MnS deposition. Using various electrochemical techniques, the effects of light illumination on charge storage performance were examined. The results showed that sample S1 retained real capacitance readings of 10 and 6.3 F g^−1^ at scan rates of 20 mV s^−1^ and *C*_d_ of 10 μA cm^−2^, respectively, before and after exposure to light. The role of extra electron–hole pairs in charge storage and the longer discharge duration made possible by light-induced carriers of charges were shown to be important variables. Furthermore, 3.0 × 3.0 cm^2^ MnS@V_2_O_5_-BiVO_4_ electrodes and MnS/graphite ASSC were constructed. These devices showed that they could power LED lights and charge under light irradiation, indicating possible real-world uses for them as effective energy storage devices. MnS@V_2_O_5_-BiVO_4_'s remarkable performance demonstrates its potential for energy uses. Hassan and collaborators^208^ presented a brand-new carbonized wood substrate component onto which AgCo-MOF/MnS is placed to create AgCo-MOF/MnS@CWS, a composite material. The mixture has remarkable properties, such as superior *C*_sp_, flexibility, adaptability, and high electrical power generation. The composite performed admirably in a three-cell assembly test, providing a *C*_sp_ of 1068C g^−1^ at 1.0 A g^−1^. After 12 000 cycles, stability tests showed that this design could maintain up to 98% of its original capacity. Furthermore, AgCo-MOF/MnS showed a 45 mV per dec Tafel slope and a 146 mV overpotential.

### 5.2. Nickel-based electrode

The use of nickel sulfide (NiS) and its cobalt-doped form, NiCo_2_S_4_, has gained traction in energy storage and supercapacitor (SC) applications. The electrochemical properties of these TMSs are desirable, showing high *C*_sp_ and excellent rate capability. The special structures and compositions of NiS and NiCo_2_S_4_ materials hold promise for better energy storage devices and for boosting sustainable energy technology.

#### 5.2.1. NiS

Wood-based hollow carbon spheres (WHCSs) were created by R. Guo and associates^213^ through the liquefaction of wood and subsequent emulsification, curing, carbonization, and activation procedures. The surface of these microspheres was subsequently hydrodeposited with NiS to produce NiS/WHCS, a material for supercapacitor electrodes. The resultant NiS/WHCS microspheres had a substantial total pore capacity of 0.14 cm^3^ g^−1^, specific surface area of 307.55 m^2^ g^−1^, and a flower-like shape with a core–shell structure. These results show that NiS/WHCS has a high *C*_sp_ and remarkable durability, indicating its potential as an electrode material for SCs.

**Fig. 8:**
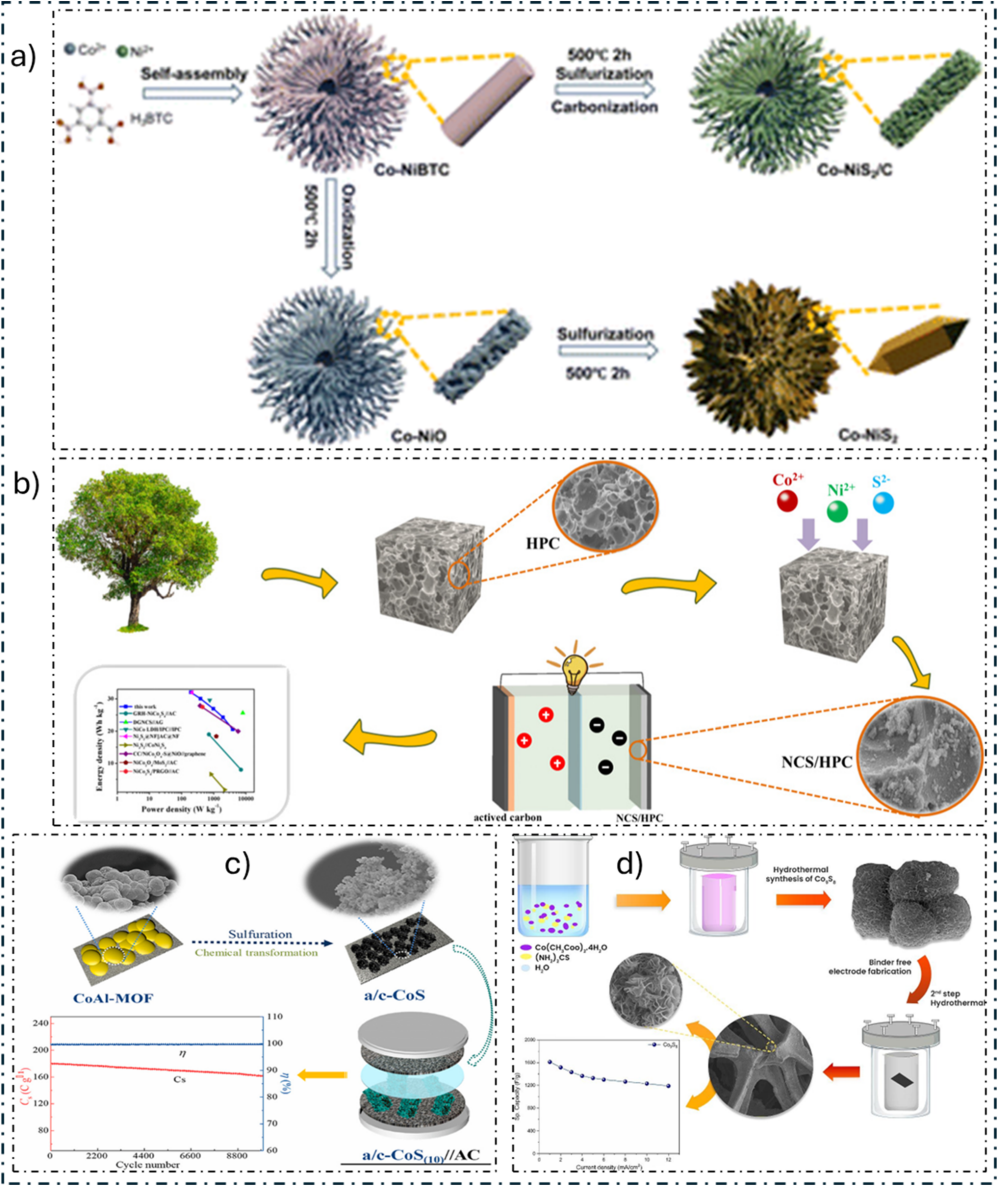
Innovative architectures of transition metal sulfide (TMS) electrodes for enhanced supercapacitor performance. This figure highlights key structural designs: (a) an urchin-like Co-NiS_2_/C precursor-derived electrode maximizing active sites. (b) NiCo_2_S_4_ nanoparticles grown on lignin-derived hierarchical porous carbon (HPC) for superior conductivity and stability. (c) An amorphous/crystalline CoS heterointerface boosting redox activity. (d) Binder-free, flower-like Co_9_S_8_ nanosheets on nickel foam, increasing surface area and ion diffusion. These architectures exemplify strategies like defect engineering, carbon hybridization, and binder-free design to optimize TMS electrode performance.^209–212^

A new micro-flower-like NiS structure was created by W. Wei and associates,^214^ consisting of ultra-thin NSs that resemble graphene and feature many flaws as the basic building blocks. This synthesis was accomplished using economical sodium chloride (NaCl) as a dispersion and friction agent in an inventive solvent-free compound–direct reaction approach. This method controlled the development direction of NiS and guaranteed sufficient interaction between sulfur and nickel ions. Ultra-thin NiS NSs, which resemble graphene, efficiently shorten the distance that ions and electrons must travel. Furthermore, the number of ion adsorption and storage sites along with high-activity spots for electrode materials is increased by introducing defects in the NiS NSs. Additionally, by improving the local electronic structure, this modification facilitates charge transfer and ion diffusion. Trimesic acid was employed as an organic ligand in a coprecipitation process by Hu and colleagues^209^ (Fig. 8a), who used a 3D urchin-like Co-NiBTC NRs matrix as the precursor to synthesize Co-NiO, Co-NiS_2_, and Co-NiS_2_/C. With increased sulfur vacancies and pores from one-step pyrolysis, Co-NiS_2_/C preserves a stable urchin-like shape inherited from the predecessor according to theoretical and experimental research. The density of free electrons is increased by co-doping, and electron transmission is improved by the carbon matrix. Its comparatively large surface area, which reveals electrochemically active spots, its porous structure, which facilitates ion transit, and its binding of a carbon skeleton, which increases electrical conductivity, are the sources of these exceptional electrochemical capabilities. These results provide insights into how to construct different metal compound materials generated from MOFs and introduce vacancies for the electrochemical storage of energy.

#### 5.2.2. NiCo_2_S_4_

J. Zhao and colleagues^215^ created a NiCo_2_S_4_@NiCo(HCO_3_)_2_ core–shell heterostructure (NiCo_2_S_4_@HCs) in which cross-linked NiCo_2_S_4_ nanowires intricately join NiCo(HCO_3_)_2_ polyhedrons. This purposeful design reduces volume expansion throughout cycles of charging and discharging, in addition to increasing the number of electroactive spots. Moreover, DFT calculations confirm that the NiCo_2_S_4_@HCs heterostructure enhances efficient and rapid redox reactions *via* facilitated OH^−^ adsorption/desorption and accelerated intra-electrode electron transfer. Cycling NiCo(HCO_3_)_2_ leads to NiCo(OH)_2_CO_3_ and then to highly active NiCoOOH, as shown by *ex situ* X-ray diffraction and Raman spectroscopy. Exceptional electrochemical stability is achieved as a result of this novel structural design, which safely maintains the structural integrity of electrode materials and alters interface charge states.

NiMoO_4_@NiCo_2_S_4_ composites may be easily synthesized by J. Lu and co-authors^216^*via* a mix of hydrothermal and electrodeposition processes. When combined with extremely reactive NiMoO_4_, extremely conductive NiCo_2_S_4_ NRs have positive synergistic effects. At 2 A g^−1^, NiMoO_4_@NiCo_2_S_4_ exhibits an impressive *C*_sp_ of 1282.6C g^−1^, or 2565.2 F g^−1^. Furthermore, after 5000 charge–discharge cycles, NiMoO_4_@NiCo_2_S_4_ exhibits improved capacity retention from 75.93% to 84.7% compared to a NiCo_2_S_4_ electrode. Based on DFT calculations, the electrochemical performance of NiMoO4@NiCo_2_S_4_ is enhanced when the work function (WF) increases to 5.13 eV and the density of states (DOS) around the Fermi level increases considerably. Moreover, an amazing *E*_d_ of 75.3 W h kg^−1^ is attained at 800 W kg^−1^ in an ASC setup (NiMoO_4_@NiCo_2_S_4_//AC). Li and colleagues produced hierarchical porous carbon (HPC) from lignin, an ecologically acceptable enzymatic hydrolysis material^228^ (Fig. 8b). HPC materials serve as an appropriate host matrix for NPs and provide extraordinary electrochemical performance for the NP/HPC composite. A facile one-step solvothermal synthesis directly produced NiCo_2_S_4_ nanoparticles on the inner surface of a 3D lignin-derived HPC, showing impressive results. The resulting NiCo_2_S_4_/HPC composite is a promising electrode material for supercapacitors. The NiCo_2_S_4_/HPC composite shows outstanding rate performance and a high *C*_sp_ of 1264.2 F g^−1^ at 1 A g^−1^. This extraordinary characteristic is attributed to the efficient fusion of NiCo_2_S_4_ and HPC, as well as their robust chemical bonds, which enable superior electrical conductivity and many exposed electroactive sites.

Using a solvothermal technique, N. J. Panicker and co-authors^217^ created porous carbon g-C_3_N_4_ (pCCN) NSs, which were further treated with acid and calcined. NiCo_2_S_4_ was then grown on the restricted structure of the pCCN/rGO heterostructure (pCRNCS) using a simple hydrothermal technique to produce a hybrid material appropriate for the SC electrodes. Carbon self-repairing g-C_3_N_4_ (CCN) has better electronic conductivity and activity than pristine g-C_3_N_4_. This is because substitutional or interstitial carbon atoms create longer delocalized π-electrons, and acid treatment breaks larger CCN planes into smaller segments, enhancing the edge oxygen and nitrogen functional groups. The addition of porous CCN suppresses the aggregation of graphene sheets by facilitating strong electrostatic contact between GO and CCN. Because of the extended highly reactive region and defects in pCCN, as well as the 2D/2D heterostructure assembly of the high surface area of rGO, the synthesized pCRNCS electrode demonstrates an exceptionally high specific capacitance, which promotes the nucleation and confined growth of NiCo_2_S_4_ within the framework. The unique capacitive properties of the self-healing g-C_3_N_4_/rGO@NiCo_2_S_4_ porous carbon composite suggest the potential for building highly efficient energy storage devices.

### 5.3. Zinc-based electrodes

Zinc sulfide (ZnS) has good electrical conductivity, a large surface area, and advantageous redox characteristics, making it a valuable material for supercapacitors and electrochemistry. ZnS has potential properties for a range of energy storage and conversion applications due to its ability to promote increased electrochemical performance, quick electron transport, and enhanced charge storage.

N. Salah and associates^218^ employed co-precipitation and chemical oxidation polymerization techniques to produce PANI and zinc sulfide quantum dots (ZnS QDs), respectively. This work presented the first use of PANI/ZnS QD nanocomposites as SC electrodes. PANI/ZnS QDs were found to have optimum *C*_sp_ readings of 893.75 and 929 F g^−1^ at 0.5 A g^−1^ and 5.0 mV s^−1^, respectively, when the electrochemical characteristics were assessed in 1.0 M H_2_SO_4_. Furthermore, over 1000 cycles, the *E*_d_ was 178.75 W h kg^−1^, and the *P*_d_ was 300 W kg^−1^, showing a high cyclic stability of 97.8%. Because of their large surface area, ZnS QDs increased electrochemical performance by enabling better contact between PANI and the electrolyte, hence reducing the RRES of the SC electrode.

An anisotropic superstructure called ZnS/SOM-C was developed by X. Wu and colleagues^219^ using a micro-reaction technique. Interfacial C-S-Zn bonds hold ultrafine ZnS nanoclusters to a conductively organized macro-microporous carbon skeleton. Large accessible surfaces are extensively distributed to active sites, and 3D organized macro-microporous pathways are the features of this structure that improve electrolyte mass diffusion, interfacial charge transfer, and faradaic ion storage (a capacitance of 1158 F g^−1^ in the KOH electrolyte). ZnS/SOM-C is constructed into a fibrous electrode for a flexible SC using microfluidic spinning, exhibiting high capacitance (791 F g^−1^), commercial-level Ed (172 mW h g^−1^), and long-lasting stability. Thus, flexible SC shows promise in the new energy and wearable industrial sectors by enabling wearable self-powered devices, including self-cleaning ventilatory masks, smartwatches, and displays. A two-step electrodeposition approach was used by F. Tian and co-authors^220^ to synthesize sulfur-vacancy-enriched sheet-like Ni_3_S_2_@ZnS composites on a 3D network porous NF. At a *C*_d_ of 19 A g^−1^ (5 mA cm^−2^), the resultant Ni_3_S_2_@ZnS/NF demonstrated a *C*_sp_ of 1141.9C g^−1^ (241.0 mC cm^−2^). An all-solid ASC with an impressive *E*_d_ of 12.0 μW h cm^−2^ at a *P*_d_ of 7.2 mW cm^−2^ and an outstanding capacitance retention rate of 86.7% after 50 000 cycles was manufactured using the Ni_3_S_2_@ZnS/NF positive electrode. According to experimental and theoretical results, the presence of sulfur vacancies improved the electrode's hydroxide diffusion, surface activity, and adsorption capacity by adjusting the local electron concentration of the surrounding cations. This promoted faster interfacial redox reactions, which enhanced the Ni_3_S_2_@ZnS/NF electrode's exceptional electrochemical performance. This work sheds light on controllable production techniques for vacancy-rich 2D NSs and their possible uses in electrochemistry.

### 5.4. Cobalt-based electrodes

Because of its high conductivity, superior redox activity, and capacity to facilitate quick electron and ion movement, cobalt sulfide (CoS) is a material that shows great promise for use in supercapacitors. Because of its high specific capacitance and fast charging and discharging, CoS is a desirable material for energy storage applications.

The design and synthesis of an a/c-CoS with a heterointerface between the crystalline and amorphous phases were reported by Liao *et al.*^211^ This structure was created using a CoAl-MOF template and a simple sulfuration procedure. The resultant a/c-CoS_(10)_ achieved a high specific charge of 1487.0C g^−1^ at 1 A g^−1^, combined with good rate capability and cycling stability, maintaining 87.4% capacity after 5000 cycles owing to their careful adjustment of sulfur content, which maximized electrochemical performance. The amorphous-crystalline heterophase improves OH^−^ adsorption and increases electrochemical activity over crystalline Co_3_S_4_, allowing for quicker ion/electron transit and reaction kinetics according to DFT studies. Wan *et al.*^221^ used a two-step electrodeposition process to build a new heterostructure comprising NiSe/MnSe nanospheres interconnected with CoS nanosheets on a carbon paper (CP) substrate. CoS and NiSe/MnSe work in concert to improve structural stability, ion transport, conductivity, and electroactivity. The resultant hybrid supercapacitor exhibits long-term endurance with 93.3% capacity retention over 20 000 cycles at 20 A g^−1^ and a high energy density of 65.8 W h kg^−1^ at 1212.3 W kg^−1^. This work offers a simple approach for developing TMS-based electrode materials to improve the energy density and cycle stability of hybrid supercapacitors.

Halder *et al.*^212^ (Fig. 8d) created binder-free Co_9_S_8_ electrodes with a layered, flower-like shape on nickel foam using a two-step hydrothermal process. The BET study showed an enhanced surface area from 146.4 m^2^ g^−1^ to 210.9 m^2^ g^−1^, facilitating effective ion diffusion and diffusion-controlled electrochemical processes. Structural investigation using XRD, XPS, TEM, and SEM revealed comprehensive morphological features. Electrochemical performance was improved by the binder-free design, which revealed more redox-active sites. CoS nanosheets were created by Nasiri *et al.*^222^ using a metal–organic framework and applied to NF. A hybrid supercapacitor using CoS/NF as the positive electrode and activated carbon as the negative electrode demonstrates the high potential of CoS nanosheet arrays for advanced energy storage.

### 5.5. Molybdenum-based electrodes

Because of its exceptional conductivity, large surface area, and distinctive layered structure, molybdenum sulfide (MoS_2_) has drawn interest in supercapacitor research. Because of these characteristics, MoS_2_-based electrodes can have high specific capacitance and strong energy density because of fast ion diffusion and charge storage. Using hybrid materials and nanoscale engineering to overcome issues such as conductivity and stability restrictions, MoS_2_'s promise for long-lasting, high-performance supercapacitors is being increased.

**Fig. 9:**
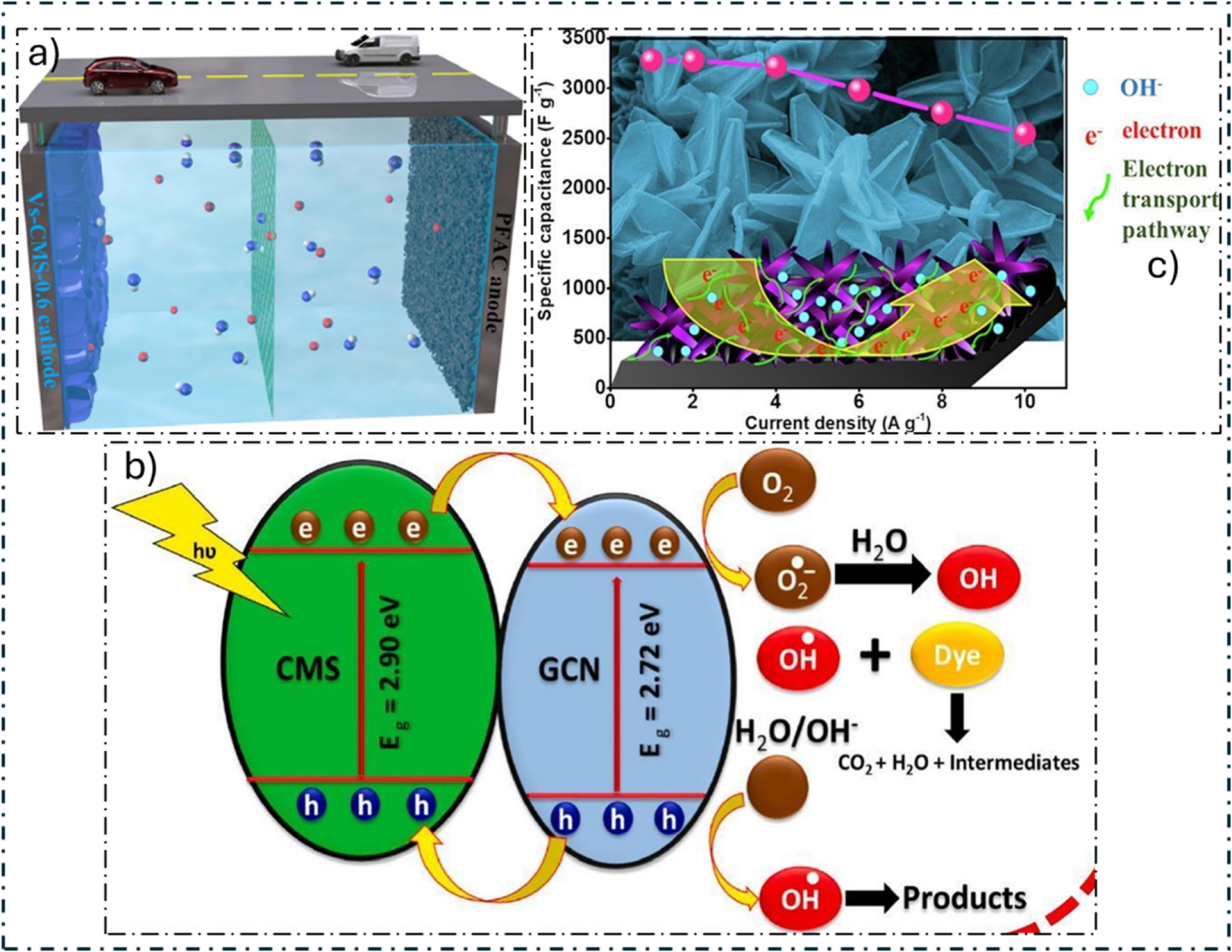
Advanced architectures of molybdenum-based sulfide (MoS_2_ and related) electrodes for high-performance supercapacitors. This figure highlights key design strategies: (a) sulfur vacancy-enriched cobalt molybdenum sulfide (Vs-CMS) nanosheets for enhanced active sites and conductivity. (b) A CoMoS_4_/g-C_3_N_4_ nanocomposite leveraging synergistic effects for improved capacitance and stability. (c) A stellate-shaped Co_3_S_4_-Mo_15_S_19_ hybrid derived from a ZIF-67 template, offering a high surface area and robust structure. These designs focus on defect engineering, hybridization with conductive supports, and unique morphologies to maximize electrochemical performance.^223–225^

Li *et al.*^223^ (Fig. 9a) used hydrothermal and NaBH_4_ reduction techniques to create sulfur vacancy-reinforced cobalt molybdenum sulfide nanosheets (Vs-CMS) as integrated cathodes. Faster electron and ion transport is encouraged by the addition of sulfur vacancies, which increase the number of active sites for faradaic redox processes. Pathak *et al.*^226^ created a NiMo_3_S_4_/black phosphorus (BP) hybrid using a straightforward one-step *in situ* hydrothermal process. Following the improvement, the NiMo_3_S_4_/BP hybrid outperformed pure NiMo_3_S_4_ with a high specific capacitance of 830 F g^−1^ at 1 A g^−1^. Because of the higher electronic states close to the Fermi level, the DFT simulations showed that the NiMo_3_S_4_/BP hybrid structure has improved conductivity and quantum capacitance, which contribute to greater charge storage capabilities.

To investigate the potential of this cobalt molybdenum sulfide-graphitic carbon nitride (CoMoS_4_/g-C_3_N_4_) nanocomposite for photocatalytic applications and as an electrode material for energy, Kamran *et al.*^224^ (Fig. 9b) synthesized it. Vibrational modes and porosity were among the important features of the nanocomposite discovered by structural investigation using XRD, Raman, and SEM. As the CoMoS_4_ concentration increased, the composite's energy band gap decreased from 2.54 to 2.44 eV. After 4000 cycles, the material retained 88% of its original capacitance, demonstrating exceptional cycling stability. Using one-pot hydrothermal sulfurization, Saha *et al.*^225^ chemically synthesized a leaf-like ZIF-67 as a sacrificial template (Fig. 9c), resulting in a Co_3_S_4_-Mo_15_S_19_ (CMS/NF) cobalt–molybdenum hybrid sulfide with a characteristic stellate-shaped architecture. With 77.7% retention at 10 A g^−1^, the CMS/NF hybrid showed an exceptional specific capacitance of 3283 F g^−1^ at 1 A g^−1^. In an asymmetric supercapacitor, a KOH electrolyte yielded an energy density of 40.8 W h kg^−1^ at a power density of 400 W kg^−1^ and displayed exceptional cycling stability, retaining 81% of its initial capacity after 5000 cycles with a coulombic efficiency exceeding 100%. The design of high-performance supercapacitor electrodes was supported by DFT simulations, which verified enhanced conductivity introduced by more electronic states close to the Fermi level and calculated quantum capacitance that matched the experimental findings.

## 6. Outlooks and future perspective

To summarize, the exceptional SC effectiveness and high cyclic stability can be credited to the following factors: the development of a unique morphology with efficient electrochemistry, the absence of binders, an *in situ* growth technique, and the use of an easy-to-understand manufacturing and synthesis method. These factors represent a significant improvement over the previous study. These results provide new insights into the design and construction of electrodes for supercapacitors, which are energy storage devices, as well as the selection and synthesis of materials.

### 6.1. Challenges of SCs

#### 6.1.1. Technical problems

*E*_d_ of supercapacitors is low. Enhancing energy density is still a major goal in the development of supercapacitors. Technological and manufacturing improvements can boost supercapacitor capacity; however, long-term solutions depend on developing superior electrode and electrolyte materials with better electrochemical performance. Current supercapacitors are less compact because of their poor energy density, making them large devices. To boost the energy densities in double-layer capacitors, researchers have focused on expanding the working voltage window, enlarging the surface area of the electrode materials, or a combination of both the approaches. The main aims of ongoing studies are to synthesize novel materials boasting large surface areas and to employ suitable organic electrolytes that function across wider voltage ranges. Supercapacitors could match the energy densities of batteries if their issues are resolved.

#### 6.1.2. Establishment of an electrical model

In many cases, the supercapacitor model closely approximates the optimal model. However, less-than-ideal traits could pose substantial risks in military applications, particularly for satellite and spacecraft power systems—a concern that should not be overlooked. Due to their low energy, filters, and energy storage, all capacitors have resonance problems that may be effectively handled, but supercapacitors pose special difficulties because of their high energy density and ability to supply energy instantly. Thus, to assure system stability, supercapacitors must be designed with dependability in mind. Their effects on load characteristics, load variations, external surroundings, and potential inadvertent disturbances must be carefully studied.

#### 6.1.3. Consistent detection

Because supercapacitors have a relatively low rated voltage, numerous units must be used in series for practical applications. Maintaining a constant voltage across individual capacitors in a series connection is essential because of the high current needed for charging and discharging in these applications and the negative consequences of overcharging on the capacitor lifespan. Because supercapacitors have a relatively low rated voltage (less than 2.7 V), numerous units must be used in series for practical applications. Maintaining a constant voltage across individual capacitors in a series connection is essential because of the high current needed for charging and discharging in these applications and the negative consequences of overcharging on the capacitor lifespan.

#### 6.1.4. Industrial standards

Over a short period, supercapacitors have experienced tremendous growth, with businesses in the field ranging in their level of knowledge. Effective industry and market regulation, focusing on creating workable industry, national, and even worldwide standards, is essential to the sustainable evolution of supercapacitors as a unique energy storage technology. For supercapacitors, a thorough technical standard system should be created that addresses topics such as terminology, naming and classification conventions, electrical performance testing procedures, safety requirements, electrode material, general specifications, electrolyte standards, charged standards, production procedures, transportation guidelines, and recycling and disposal requirements. For example, the general requirements for the management and storage of supercapacitor cells and modules during scrap disposal, including the disassembly of individual cells and modules, recycling processes, electrolyte and capacitor shell treatment, plate processing, and diaphragm handling, are intended to guide and standardize the industry. These measures aim to achieve low-cost, environmentally friendly recycling and disposal practices. Implementing such standards is essential for fostering the sustainable growth of the supercapacitor industry.

### 6.2. Future perspectives

#### 6.2.1. Flexible devices

With the rapid growth of portable electronics and the rise of wearable technology, flexible energy storage devices have gained significant interest among researchers. Developing compact, flexible energy storage solutions with high electrochemical performance is of great importance. However, traditional supercapacitors are limited by the rigidity of their electrodes, which constrains their shape adaptability. Moreover, the use of metal collectors and bonding agents in electrode fabrication further diminishes the electrochemical efficiency of supercapacitors. Therefore, the next generation of flexible energy storage devices will focus on creating flexible supercapacitors that are well suited to portable electronic products. Flexible positive and negative electrodes, diaphragms, electrolytes, current collectors, and packaging materials make up flexible supercapacitors, as opposed to conventional rigid ones. This adaptability increases their potential for use in flexible and wearable technology domains by enabling them to be put together into thin, light, and intelligent designs of any size. From the standpoint of applied research, the creation of suitable flexible electrodes is crucial to the effective manufacturing of flexible supercapacitors. The creation of flexible electrodes with great performance has therefore been the focus of this field's study. Flexible electrode and flexible supercapacitor research have developed into a broad and complex field to date. The produced supercapacitors and flexible electrodes exhibit a wide range of functional characteristics and physical shapes, demonstrating the technology's rich diversity and promise.

#### 6.2.2. Improvement in the cost performance

The main and most important element for every industry's long-term success is improving product performance while reducing manufacturing costs. The development of supercapacitors necessitates not only improving manufacturing methods and procedures but also locating stable and effective electrode and electrolyte materials to increase performance while decreasing prices. One of the field's main areas of interest is this research area. For example, a U.S.-based business called Full Power Technologies is now focused on creating inexpensive ultracapacitors. In order to achieve low costs, one must (1) identify new low-cost raw materials, such as natural mineral resources; (2) look for a combination of high- and low-priced raw materials to achieve complementary performance and low overall price; and (3) enhance the production process (*e.g.*, simplifying the process) and production equipment; consequently, this will also be the primary focus and strategic objective of the product's subsequent development; (4) pay attention to the potential of the materials for industrialization and the financial concerns surrounding the use of novel electrode materials, including carbon fiber graphene; (5) research on matching existing electrode materials, such as matching existing electrode materials with the electrolyte; (6) research on group modules focusing more on the management system, capacitors, and overall service life characteristics to improve security and dependability. In summary, the advancement of science and technology, as well as the need for applications, are inextricably linked to the development of supercapacitors. We believe that the development of supercapacitors will proceed more quickly and extensively as new energy vehicles and smart wearable technology gain popularity.

#### 6.2.3. Intelligent devices

Multifunctional electrochemical energy storage devices that are intelligent and controlled are becoming more and more in demand as intelligent electronic gadgets develop quickly. Intelligent features of these devices allow both users and manufacturers to configure them for various purposes that are suited to practical requirements. We may expect the development of adaptable gadgets that improve user-friendly, customized interactions with wearable and bio-integrated electronics by creating new materials, creating novel architectures, and utilizing supercomputer simulations and artificial intelligence. In the future, it will be very desirable to incorporate supercapacitors with characteristics such as shape memory, electrochromic qualities, and even self-healing capabilities.

## 7. Conclusion

We examined current developments and difficulties in the field of metal oxide and metal sulfide-based supercapacitor electrodes in this review. After a thorough review of the literature, several important discoveries were made that have shaped the direction of SC technology. Because of their proven technology and innate durability, metal oxide electrodes have long been the mainstays in the industry. Well-known examples, such as RuO_2_, have shown remarkable capacitance but at the cost of using rare earth elements, which present financial challenges. Ongoing studies, however, on other oxide materials, such as MnO_2_, present viable paths for improving cost-effectiveness without sacrificing performance measures. On the other hand, metal sulfide electrodes have become strong competitors due to their high theoretical capacitance and significant developments in materials such as NiCo_2_S_4_. Although there are stability issues and difficulties with the synthesis process, incredible advancements in sulfide-based electrodes highlight their potential to completely transform the supercapacitor industry. It is clear from navigating this shifting terrain that there are many facets to the search for the perfect supercapacitor material. Although metal sulfides and oxides have unique benefits and drawbacks, the creation of composite materials might be the key to obtaining improved performance attributes. Furthermore, the growing investigation of metal nitrides offers an exciting opportunity to broaden the range of electrode materials; nevertheless, additional studies are necessary to confirm their effectiveness. Future studies should concentrate on improving the performance, stability, and synthesis of electrodes made of metal oxide and sulfide. Furthermore, the investigation of new electrode materials and improvements in electrode architecture and electrolyte formulations will spur the development of supercapacitor technology in the direction of increased cost-effectiveness, long-term viability and effectiveness. This analysis concludes by highlighting the critical role that metal oxide and sulfide-based electrodes have played in pushing the boundaries of SC technology. We are prepared to usher in a new era of energy storage solutions that will encourage innovation and sustainability in the worldwide quest for a greener future by embracing multidisciplinary cooperation and using the synergistic potential of varied materials. The options for material design have been further increased by the insertion of binary metal oxides and composites, which are accomplished by adding more compounds and a second transition metal. Transition metal oxides have enormous potential for increasing electrolytes, encouraging efficient electrochemical redox processes, and ultimately leading to significant improvements in supercapacitor performance through the application of nanoarchitecture and rational design. By employing binary metal oxides and innovative nanostructured metal oxide-based electrodes, the supercapacitor industry can make significant strides in energy density, cycling stability, environmental sustainability, flexible-wearable electronics, 3D designs, and self-powered systems. This encompasses large-scale production as well as improvements in electrolytes and manufacturing techniques. The optimisation of microstructure, hybrid system composition, electrical conductivity, mechanical integrity, and the use of advanced characterization techniques to understand the electrochemical mechanisms at play and correlate them with overall performance will all help to develop reliable and efficient metal oxide-based supercapacitors that are appropriate for specific applications.

## Conflicts of interest

There are no conflicts to declare.

## Data Availability

No primary research results, software or code have been included and no new data were generated or analysed as part of this review.
